# *N*-Acetyltransferase 2 Inhibits Myopia by Maintaining Mitochondrial Metabolism in Scleral Fibroblasts

**DOI:** 10.34133/research.0969

**Published:** 2025-10-29

**Authors:** Wei Gong, Zhi Shen, Lingyi Zhao, Tianyu Cheng, Bo Zhang, Jun Chen, Jingjing Wang, Zhiqiang Li, Xuemin Jian, Yongyong Shi, Yanqin Wen, Xun Xu, Xiangui He

**Affiliations:** ^1^ Shanghai Eye Diseases Prevention & Treatment Center/Shanghai Eye Hospital, School of Medicine, Tongji University, Shanghai, China.; ^2^ Department of Ophthalmology, Shanghai General Hospital, Shanghai Jiao Tong University School of Medicine, National Clinical Research Center for Eye Diseases, Center of Eye Shanghai Key Laboratory of Ocular Fundus Diseases, Shanghai Engineering Center for Visual Science and Photomedicine, Shanghai, China.; ^3^ Department of Cardiology, Renji Hospital, School of Medicine, State Key Laboratory for Oncogenes and Related Genes, Shanghai Cancer Institute, Shanghai Jiao Tong University, Shanghai, China.; ^4^ State Key Laboratory of Medical Neurobiology and MOE Frontiers Center for Brain Science, Institutes of Brain Science, Fudan University, Shanghai, China.; ^5^ Bio-X Institutes, Key Laboratory for the Genetics of Developmental and Neuropsychiatric Disorders (Ministry of Education), Collaborative Innovation Center for Brain Science, Shanghai Jiao Tong University, Shanghai, China.; ^6^ Institute of Neuroscience, Chinese Academy of Science, Center for Excellent Brain Science and Intelligence Technology, Chinese Academy of Sciences, Shanghai, China.

## Abstract

The excessive elongation of eye axis is a primary contributing factor of myopia, yet the underlying genetic mechanisms remain not fully understood. The study was to explore risk-associated genes of myopia and identified that *N*-acetyltransferase 2 (NAT2) acted as a regulator in the pathological process of myopia. The genome-wide association study analysis was performed for axial length of eyes in a Chinese population aged 4 to 18 years (*n* = 6,345), and the most significant locus was 8p22, with *NAT2* identified as a potential risk-associated gene for myopia. NAT2 was down-regulated by hypoxia in form deprivation-induced murine myopia, and the adeno-associated virus-induced genetic intervention of NAT2 in sclera influenced myopia progression. For mechanism, NAT2 could regulate the phenotypic transition and extracellular matrix remodeling of scleral fibroblasts by maintaining mitochondrial metabolism and inhibiting the ROS/TGF-β pathway, which also had effects on choroidal vascular function. Additionally, administration with α-ketoglutarate, a downstream metabolite of NAT2, effectively suppressed myopia progression in murine models. These findings highlight NAT2 as a critical regulator of myopia pathogenesis, offering new insights into potential therapeutic strategies targeting mitochondrial metabolism.

## Introduction

Myopia is becoming a critical global health concern, with its prevalence rising markedly in recent decades—particularly among children and adolescents [1,2]. Axial myopia, driven by the excessive elongation of eyeballs, remarkably elevates the risk of severe blinding complications such as maculopathy [3,4]. However, current control strategies are insufficient to cope with the growing myopia epidemic, underscoring the necessity for exploring novel mechanistic insights and intervention targets of myopia.

A key driver of myopia progression lies in the dynamic remodeling of scleral extracellular matrix (ECM) and scleral fibroblast phenotype [5,6]. It is well established that choroidal vascular dysfunction-induced hypoxia could destroy the stability of scleral ECM in myopic eyes [6–10], which is important to the structural integrity of eyeballs. Scleral ECM remodeling leads to biomechanical weakening of sclera and progressive elongation of axial length (AL) [8,11]. Notably, the scleral ECM is primarily synthesized by scleral fibroblasts, whose phenotypic transition to myofibroblasts is a crucial event in myopia pathogenesis [12]. The dysfunction of scleral fibroblasts in myopia not only alters cellular behavior and ECM composition but also up-regulates matrix metalloproteinases (MMPs), exacerbating ECM degradation and scleral thinning [13,14]. Consequently, targeting fibroblast phenotypic transition and ECM remodeling represents a promising therapeutic strategy for myopia.

The formation and progression of myopia is determined by multiple factors, among which genetic factors play important roles [1]. Extensive genomic analyses, particularly genome-wide association studies (GWAS), have been widely utilized to identify genetic factors linked to myopia susceptibility [15–19]. Our ongoing GWAS, focusing on AL in a youth cohort, has implicated *N*-acetyltransferase 2 (NAT2), a vital metabolic enzyme, as a potential modulator of myopia progression. NAT2 has been proved to be a regulator of mitochondrial function [20] and associated with metabolism-related diseases, such as diabetes [21,22]. Notably, mitochondrial metabolism is involved in several ocular diseases, like dry eye disease, external ophthalmoplegia, and retinopathy [23,24], but its association with myopia has rarely been investigated. Thus, the present study aims to validate *NAT2* as a myopia risk-associated gene and delves into its impact on scleral fibroblasts in myopia probably by regulating mitochondrial metabolism, which will provide novel insights and therapeutic avenues for the management of myopia.

## Results

### *NAT2* is identified as a risk-associated gene of myopia

**Fig. 1.**
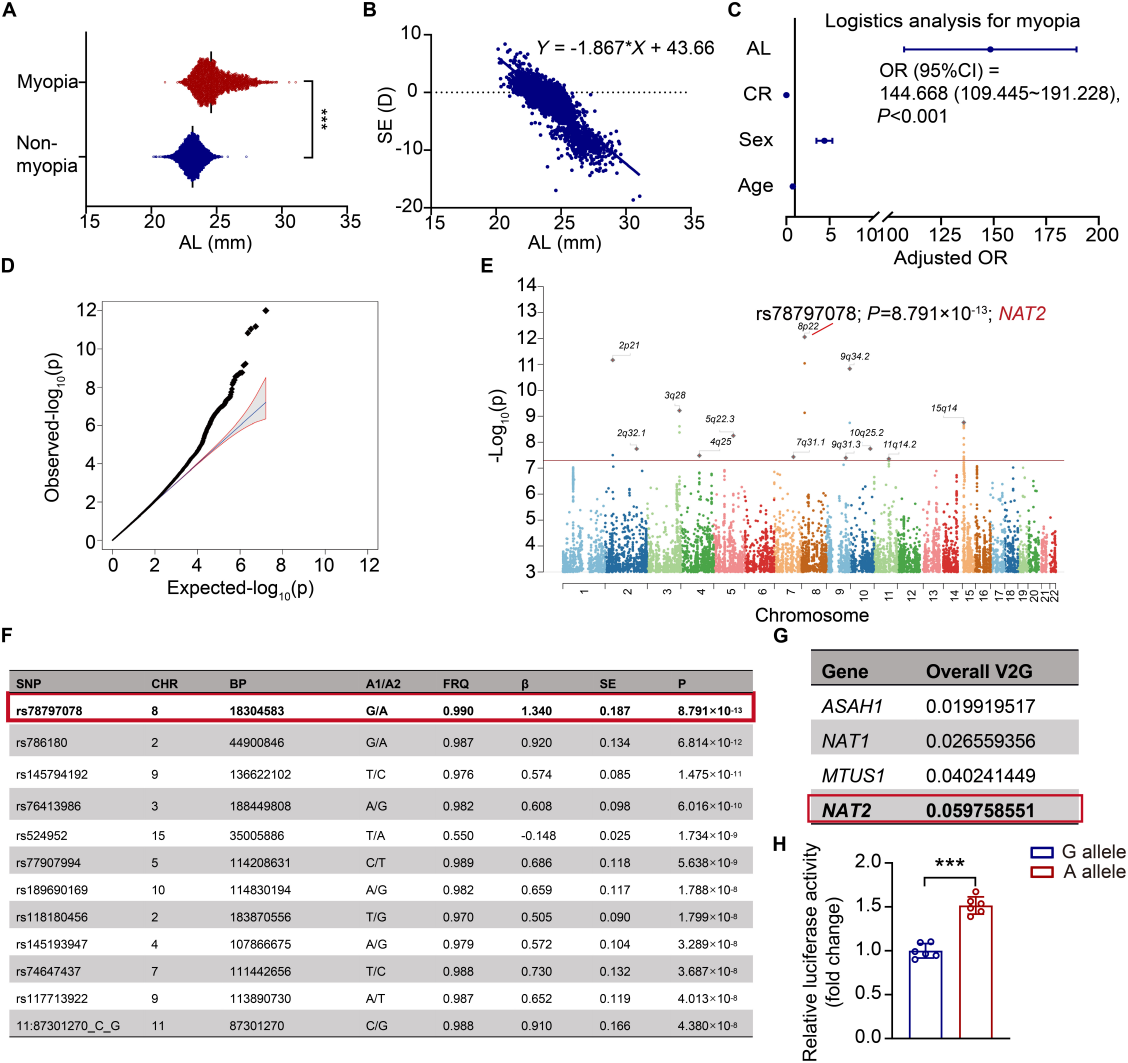
*NAT2* is a risk-associated gene of myopia. (A) Distribution of AL in population with or without myopia. ***P < 0.001 for the comparison of AL between population with or without myopia. (B) Linear regression analysis of AL and spherical equivalent (SE) in the population. (C) Logistic regression analysis for myopia. (D) Quantile–quantile plot of the GWAS analysis displaying the observed significance versus the expected significance for each variant. (E) Manhattan plot of the GWAS analysis. The *x* axis shows the genomic position, and the *y* axis shows the significance of SNPs on a −log_10_ scale. The red line represents the threshold for genome-wide significance (5 × 10^−8^). (F) Genome-wide significant loci for AL. (G) Open Targets Genetics analysis on candidate genes. (H) Human scleral fibroblasts were cotransfected with pGL3-SNP rs78797078 (G allele/A allele) (±200 base pairs)-SV40-Luci for 48 h. Data are expressed as mean ± SD.

A total of 6,345 participants (52.3% male, *n* = 3,320) aged 4 to 18 years (mean age: 9.59 ± 1.61 years) were included in the cohort analysis and GWAS. The ophthalmic characteristics of the cohort were shown in Table S1. Compelling evidence confirms that AL serves as the primary structural driver of myopic progression, directly contributing to scleral thinning and retinal pathology [25]. Consistently, in the current cohort, AL was proved to be significantly correlated with the degree and occurrence of myopia (Fig. 1A to C). To identify the risk-associated genes of myopia, we conducted GWAS for AL in the cohort and a total of 8,101,202 variants were analyzed, revealing 12 genome-wide significant loci (*P* < 5 × 10^−8^; Fig. 1D to F). Almost all these index single-nucleotide polymorphisms (SNPs), except for the variant rs524952, exhibited low frequency (0.01 to 0.05) in our sample. Five of them (rs524952 [26–29], rs189690169 [16,27,28,30], rs118180456 [27], rs786180 [16], and rs74647437 [31]) were previously reported to be associated with myopia-related traits or are located next to the previously identified SNPs (± 250 kb).

The SNP with the leading significance was rs78797078 [β = 1.340 (SE = 0.187) for the G allele, *P* = 8.791 × 10^−13^], and its nearest gene encodes *NAT2*. *NAT2* was also predicted to be the most compelling candidate gene by Open Targets Genetics (Fig. 1G). Because of its low frequency, the effect of rs78797078 [minor allele frequency (MAF) = 0.01] on the regulation of gene expression was not assessed in the GTEx project. Therefore, we explored its proxy, rs9644606 (MAF = 0.154), which is associated with AL of the eyes at β = 0.0713 (SE = 0.0345) for the C allele. The rs9644606 C allele was significantly associated with lower expression of *NAT2* (*P* = 2.35 × 10^−5^) in the testis according to the GTEx project. Linkage disequilibrium analysis suggested that the rs78797078 G allele was related to lower expression of *NAT2*. Consistently, compared with rs78797078 A allele, G allele repressed expression of downstream gene (Fig. 1H). Considering these results and given that NAT2 is a regulator for several pathophysiological processes associated with myopia [32,33], we proposed that low expression of *NAT2* was correlated with long AL and, thus, a potential risk for myopia.

### Hypoxia contributes to the down-regulation of NAT2 in myopic sclera

**Fig. 2.**
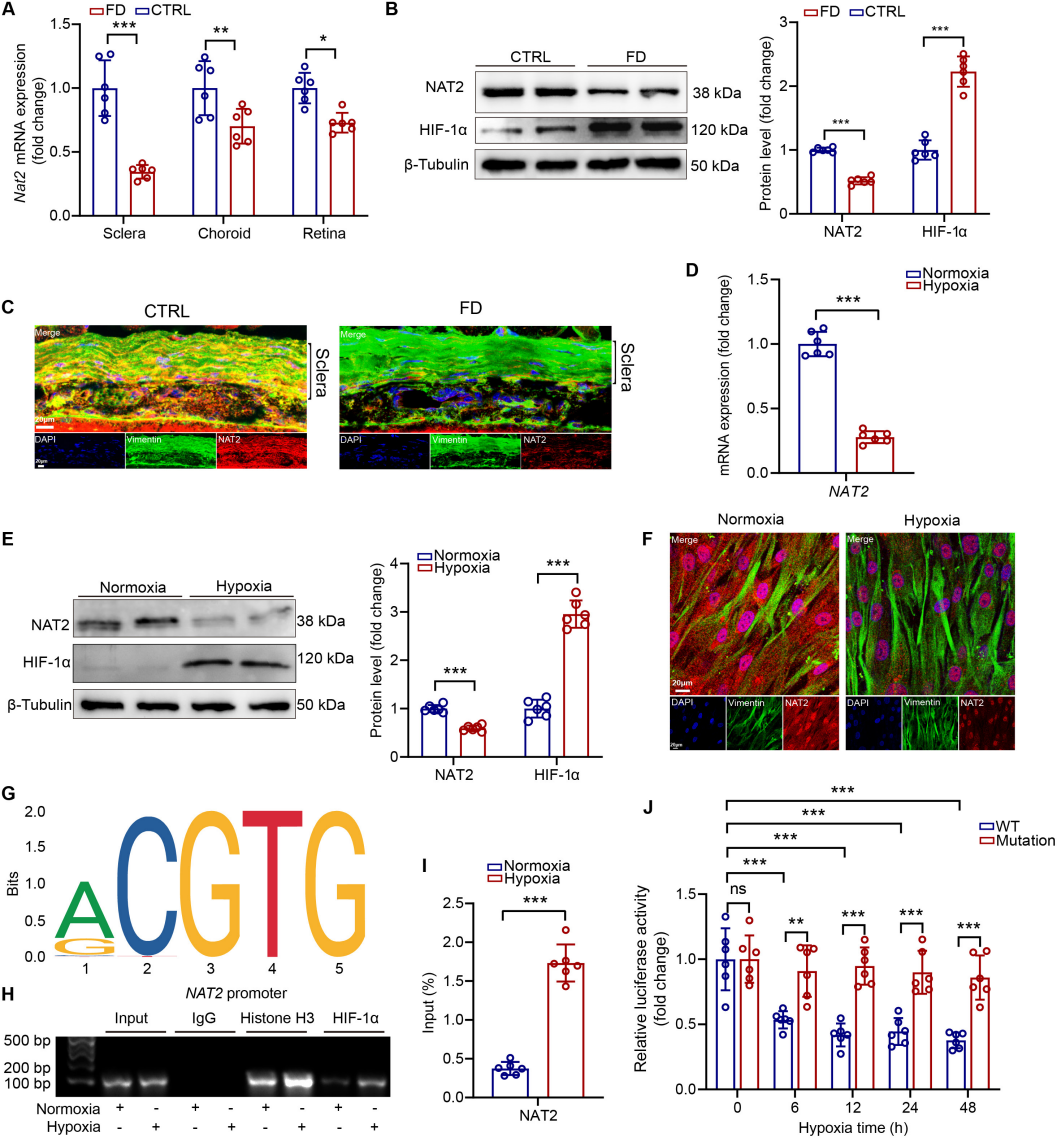
Hypoxia-induced HIF-1α contributes to the down-regulation of NAT2 in myopic sclera. (A) Quantification of *Nat2* mRNA expression measured by RT-qPCR in murine eye tissues with/without form deprivation. (B) NAT2 and HIF-1α protein expression assessed by Western blot in murine sclera with/without form deprivation (*n* = 6 per group). ***P < 0.001. (C) Representative images of NAT2 expression by immunofluorescence staining of murine sclera with/without form deprivation. (D) Quantification of *NAT2* mRNA expression measured by RT-qPCR in human scleral fibroblasts with/without hypoxia stimulation (*n* = 6 per group). (E) NAT2 and HIF-1α protein expression assessed by Western blot in murine sclera with/without form deprivation (*n* = 6 independent experiments). ***P < 0.001. (F) Representative images of NAT2 expression by immunofluorescence staining of human scleral fibroblasts with/without hypoxia stimulation. (G) Motif of HIF-1α binding NAT2 promoter, prompted by JASPAR (http://jaspar.genereg.net/). (H) ChIP-qPCR was performed with antibodies to HIF-1α, and the target promoter region of *NAT2* was amplified by qPCR. (I) Quantification of ChIP-qPCR (*n* = 6 independent experiments). ***P < 0.001. (J) Human scleral fibroblasts were cotransfected with pGL3-h_wt*NAT2*-promoter-Luci (WT) or pGL3-h_mut*NAT2*-promoter-Luci plasmid (Mutation) for 48 h and subjected to hypoxia (*n* = 6 independent experiments). Data are expressed as mean ± SD.

To further identify the relationship of NAT2 and myopia, we measured the expression of NAT2 in murine eyes with form deprivation-induced myopia (FDM), compared with those without form deprivation. We found that the most significant decrease of *Nat2* mRNA expression occurred in sclera among the tissues of myopic murine eyes (Fig. 2A). The down-regulation of NAT2 protein in myopic sclera was also verified (Fig. 2B and C). Notably, according to the immunofluorescence staining, NAT2 was mainly located in fibroblasts marked by vimentin (Fig. 2C). Since scleral hypoxia is a critical precipitating factor for myopia [5,6,13], we further performed hypoxia simulation on scleral fibroblasts to simulate the pathological microenvironment of myopia and found a decrease in the expression of NAT2 (Fig. 2D to F). The findings suggested that low expression of NAT2 was associated with formation of myopia.

Along with the down-regulation of NAT2, there was an increase in the expression of hypoxia inducible factor-1α (HIF-1α) in myopic sclera (Fig. 2B and E), which was reported as a crucial transcription factor in hypoxic response and involved in myopia pathogenesis [8,34]. Through the online tool JASPAR (http://jaspar.genereg.net/), we found that HIF-1α may bind to the ACGTG sequence within the antisense strand of *NAT2* promoter (Fig. 2G), which was consistent with the typical hypoxia response element sequence (A/G)CGTG [35]. The binding of HIF-1α on *NAT2* promoter was verified by chromatin immunoprecipitation (ChIP)–polymerase chain reaction (PCR) (Fig. 2H and I). Further, dual luciferase reporter assays revealed that transcriptional activity of luciferase plasmids carrying the wild-type NAT2 promoter was more active under hypoxia, while the mutant groups failed to induce luciferase activity (Fig. 2J). Overall, these findings illustrated that NAT2 was the direct trans-repression target of HIF-1α in scleral fibroblasts under hypoxia, which suggested the mechanism that NAT2 was down-regulated in myopia.

### Genetic intervention of NAT2 in sclera influences myopia

**Fig. 3.**
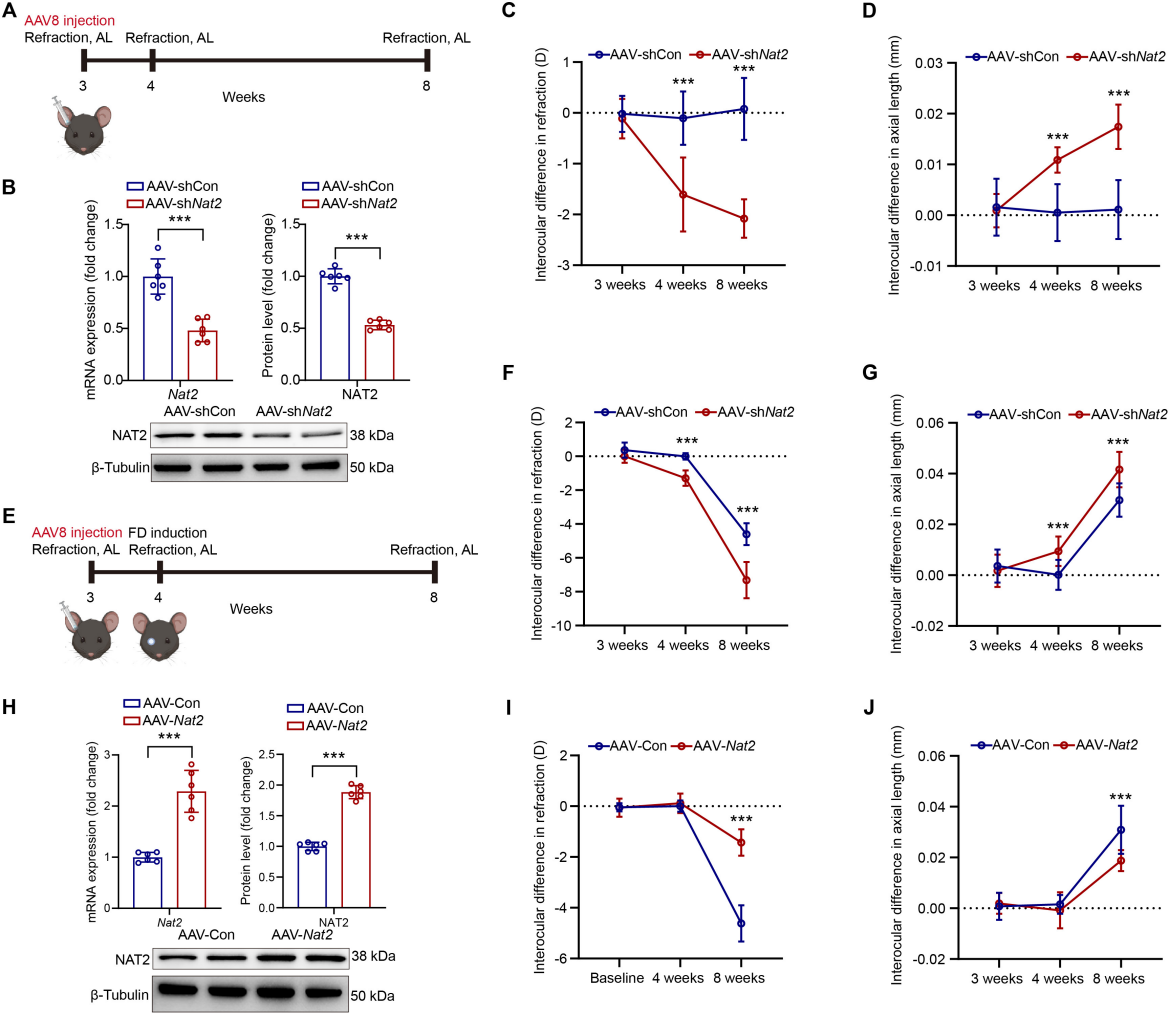
Genetic intervention of NAT2 in sclera influences myopia. (A) Genetic intervention of NAT2 in murine sclera. (B) Quantification of *Nat2* mRNA and NAT2 protein expression measured by RT-qPCR and Western blot in murine sclera with AAV-shCon or AAV-sh*Nat2* treatment (*n* = 6 per group). ***P < 0.001. (C) Interocular difference in refraction of mice with AAV-shCon or AAV-sh*Nat2* treatment (*n* = 6 per group). ***P < 0.001. (D) Interocular difference in AL of mice with AAV-shCon or AAV-sh*Nat2* treatment (*n* = 6 per group). ***P < 0.001. (E) Genetic intervention of NAT2 in murine sclera and FDM induction in mice. (F) Interocular difference in refraction of FDM mice with AAV-shCon or AAV-sh*Nat2* treatment (*n* = 6 per group). ***P < 0.001. (G) Interocular difference in AL of FDM mice with AAV-shCon or AAV-sh*Nat2* treatment (*n* = 6 per group). ***P < 0.001. (H) Quantification of *Nat2* mRNA and NAT2 protein expression measured by RT-qPCR and Western blot in murine sclera with AAV-Con or AAV-*Nat2* treatment (*n* = 6 per group). ***P < 0.001. (I) Interocular difference in refraction of FDM mice with AAV-Con or AAV-*Nat2* treatment (*n* = 6 per group). ***P < 0.001. (J) Interocular difference in refraction of FDM mice with AAV-Con or AAV-*Nat2* treatment (*n* = 6 per group). ***P < 0.001.

Adeno-associated virus (AAV) is a promising choice for gene therapy in ocular diseases [36]. To verify the effect of NAT2 on myopia development, we designed AAV8 to alter the expression of NAT2 in murine sclera. The right eyes’ sclera of 3-weeks-old mice was infected with AAV8 through sub-Tenon’s capsule injection and incubated for 1 week. We explored whether NAT2 knockdown in sclera could induce myopia in mice (Fig. 3A). Five weeks after AAV injection, compared with the AAV-shCon-injected eyes, significant reduction of scleral NAT2 expression was observed in AAV-sh*Nat2*-injected eyes (Fig. 3B), along with increases in myopic shift and AL elongation (Fig. 3C and D). Further, we conducted form deprivation on mice after 1 week of AAV injection (Fig. 3E). The knockdown of NAT2 exacerbated the progression of FDM (Fig. 3F and G). On the contrary, AAV-*Nat2* administration significantly promoted the expression of NAT2 in sclera (Fig. 3H) and suppressed the development of myopia (Fig. 3I and J). In summary, genetic inhibition of NAT2 induced myopia, while up-regulation of NAT2 attenuated it. The findings indicated that NAT2 acted as a regulator in myopia progression and might be a potential therapeutic target for myopia.

### NAT2 regulates phenotypic transition of scleral fibroblast and scleral ECM remodeling

**Fig. 4.**
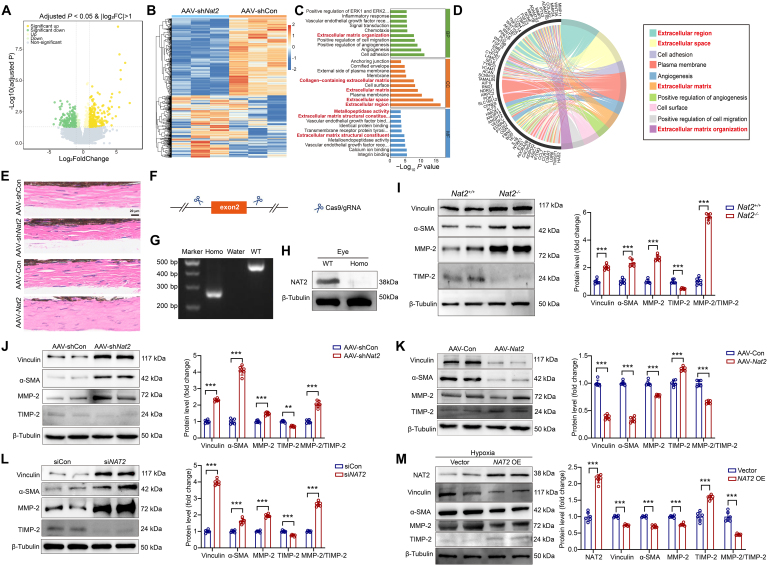
NAT2 regulates phenotypic transition and ECM remodeling of scleral fibroblasts in myopia. (A) Volcano plot reveals the magnitude and significance of differentially expressed genes between sclera from mice injected with AAV-sh*Nat2* and AAV-shCon. Differentially expressed genes were defined as genes with a Benjamini–Hochberg adjusted *P* < 0.05 and |log_2_(fold change)| > 1. (B) Heatmap of differentially expressed genes in indicated groups. Each column represents an individual replicate, and each row represents an individual gene (*n* = 3 for both groups). (C and D) Functional enrichment [Gene Ontology (GO)] analysis of genes in the intersection of the differentially expressed genes of AAV-shCon versus AAV-sh*Nat2*. (E) Hematoxylin and eosin (H&E) staining of murine scleras from indicated groups. (F to H) Establishment of *Nat2* knockout mice, and the knockout was verified with PCR and Western blot. (I) Protein expression of vinculin, α-SMA, MMP-2, and TIMP-2 in sclera of FDM mice (*Nat2^+/+^* or *Nat2*^−*/*−^) measured by Western blot (*n* = 6 per group). ***P < 0.001. (J) Protein expression of vinculin, α-SMA, MMP-2, and TIMP-2 in sclera from FDM mice with AAV-shCon or AAV-sh*Nat2* treatment measured by Western blot (*n* = 6 per group). ***P < 0.001. (K) Protein expression of vinculin, α-SMA, MMP-2, and TIMP-2 in sclera from FDM mice with AAV-Con or AAV-*Nat2* treatment measured by Western blot (*n* = 6 per group). ***P < 0.001. (L) Protein expression of vinculin, α-SMA, MMP-2, and TIMP-2 in human scleral fibroblasts with siCon or si*NAT2* treatment measured by Western blot (*n* = 6 independent experiments). ***P < 0.001. (M) Protein expression of NAT2, vinculin, α-SMA, MMP-2, and TIMP-2 in hypoxia-treated human scleral fibroblasts with/without NAT2 overexpression (OE) measured by Western blot (*n* = 6 independent experiments). ***P < 0.001. Data are expressed as mean ± SD.

To clarify the mechanism under the influence of NAT2 on myopia, we conducted RNA sequencing of sclera from FDM mice injected with AAV and the mRNA expression was compared (AAV-*shNat2* versus AAV-shCon) (Fig. 4A and B). Finally, 664 differentially expressed genes were identified and the function enrichment analysis revealed their predominant association with pathways related to ECM (Fig. 4C and D). Scleral ECM is closely associated with the structure of sclera [11], and as expected, the histochemical staining showed that the knockdown or overexpression of NAT2 altered the morphology of sclera tissue from mice with FDM (Fig. 4E). The findings indicated the role of NAT2 in regulating scleral structure in myopia.

Since the phenotype of fibroblasts is closely related to the synthesis and degradation of ECM [37,38], NAT2 might have effects on myopia by regulating phenotypic transition of fibroblasts and ECM remodeling in sclera, which are crucial processes in the pathogenesis of myopia [5]. Thus, we further explored whether NAT2 could regulate the phenotype of scleral fibroblasts. First, we constructed gene-edited mice with *Nat2* knockout (*Nat2*^−*/*−^) (Fig. 4F to H) and found that compared with the wild-type controls, deficiency of NAT2 promoted the expression of myofibroblast markers [α-smooth muscle actin (α-SMA) and vinculin] in murine sclera (Fig. 4I). Besides, MMP-2 and tissue inhibitor of metalloproteinase 2 (TIMP-2) produced by fibroblast are closely related to the ECM degradation of sclera during myopia progression [13,39,40]. After the knockout of NAT2, there was an increase in MMP-2 expression and a decrease in TIMP-2 expression, with the ratio of MMP-2/TIMP-2 raised in sclera (Fig. 4I). Consistently, NAT2 knockdown in sclera by AAV-sh*Nat2* increased the expression of myofibroblast markers and the ratio of MMP-2/TIMP-2 (Fig. 4J), while NAT2 overexpression had reversed effects (Fig. 4K).

We further conducted verification in vitro, and small interfering RNAs (siRNAs) were employed to knock down NAT2 in scleral fibroblasts (Fig. S1). The treatment of si*NAT2* led to increased expression of myofibroblast markers as well as MMP-2/TIMP-2 ratio in scleral fibroblasts (Fig. 4L), whereas NAT2 overexpression decreased them in hypoxia-stimulated scleral fibroblasts (Fig. 4M). Taken together, these findings illustrated that NAT2 regulated phenotypic transition of scleral fibroblasts and scleral ECM remodeling in myopia.

### NAT2 mediates mitochondrial metabolism homeostasis in scleral fibroblasts

**Fig. 5.**
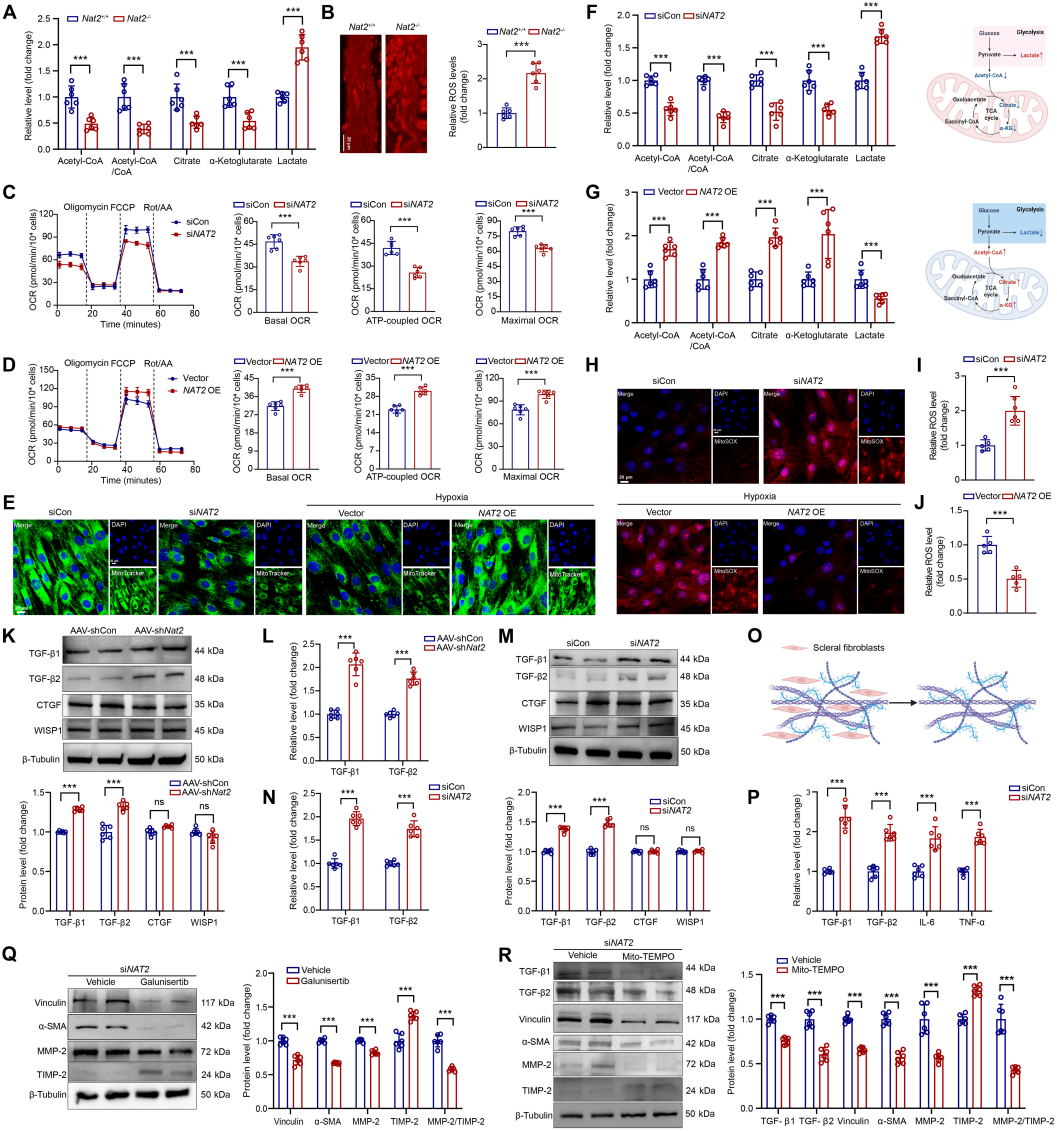
NAT2 regulates phenotype of scleral fibroblasts through the mitochondrial ROS/TGF-β pathway. (A) Level of metabolites in sclera tissues from *Nat2^+/+^* or *Nat2*^−*/*−^ mice with form deprivation (*n* = 6 per group). ***P < 0.001. (B) Dihydroethidium (DHE) staining of sclera tissues from *Nat2^+/+^* or *Nat2*^−*/*−^ mice with form deprivation (*n* = 6 per group). ***P < 0.001. (C) Mitochondrial stress test of human scleral fibroblasts with *NAT2* knockdown (*n* = 6 independent experiments). ***P < 0.001. (D) Mitochondrial stress test of human scleral fibroblasts with NAT2 overexpression (*n* = 6 independent experiments). ***P < 0.001. (E) Staining of MitoTracker in human scleral fibroblasts with *NAT2* knockdown or overexpression under hypoxia. (F and G) Measurement of metabolites in scleral fibroblasts with *NAT2* knockdown or overexpression under hypoxia (*n* = 6 independent experiments). ***P < 0.001. (H) Staining of MitoSOX in human scleral fibroblasts with *NAT2* knockdown or overexpression under hypoxia. (I and J) Level of ROS production detected by DHE assay of scleral fibroblasts with *NAT2* knockdown or overexpression under hypoxia (*n* = 6 independent experiments). ***P < 0.001. (K) Protein expression of TGF-β1, TGF-β2, CTGF, and WISP1 in murine sclera with AAV-shCon or AAV-sh*Nat2* treatment measured by Western blot (*n* = 6 per group). ***P < 0.001; not significant (ns), *P* ≥ 0.05. (L) Levels of TGF-β1 and TGF-β2 in murine sclera with AAV-shCon or AAV-sh*Nat2* treatment measured by enzyme-linked immunosorbent assay (ELISA) (*n* = 6 per group). ***P < 0.001. (M) Protein expression of TGF-β1, TGF-β2, CTGF, and WISP1 in scleral fibroblasts with siCon or si*NAT2* treatment by Western blot (*n* = 6 independent experiments). ***P < 0.001. (N) Levels of TGF-β1 and TGF-β2 in scleral fibroblasts with siCon or si*NAT2* treatment measured by ELISA (*n* = 6 independent experiments). ***P < 0.001; ns, *P* ≥ 0.05. (O) Extraction of ECM produced by scleral fibroblasts. (P) Levels of TGF-β1, TGF-β2, interleukin-6 (IL-6), and tumor necrosis factor-α (TNF-α) in ECM produced by scleral fibroblasts with siCon or si*NAT2* treatment measured by ELISA (*n* = 6 independent experiments). ***P < 0.001. (Q) Protein expression of vinculin, α-SMA, MMP-2, and TIMP-2 in human scleral fibroblasts with si*NAT2* and galunisertib treatment measured by Western blot (*n* = 6 independent experiments). ***P < 0.001. (R) Protein expression of TGF-β1, TGF-β2, vinculin, α-SMA, MMP-2, and TIMP-2 in human scleral fibroblasts with si*NAT2* and Mito-TEMPO treatment measured by Western blot (*n* = 6 independent experiments). ***P < 0.001. Data are expressed as mean ± SD.

NAT2 is a critical regulator of mitochondrial function [20], and mitochondrial metabolism is closely related to the function and phenotype of fibroblasts [41,42]; thus, we explored whether NAT2 participated in mitochondrial metabolism in scleral fibroblasts. In vivo, we found that NAT2 deficiency or overexpression altered the levels of mitochondria-related metabolites and reactive oxygen species (ROS) generation in murine sclera with form deprivation (Fig. 5A and B and Fig. S2). In vitro, energy metabolism analysis showed that altered expression of NAT2 significantly influenced the mitochondrial oxygen consumption rates (Fig. 5C and D) and the mitochondrial membrane potential (Fig. 5E) in scleral fibroblasts. These results identified the role of NAT2 in maintaining mitochondrial metabolism of scleral fibroblasts.

It is well established that the homeostasis of acetyl-coenzyme A (CoA), a key substrate of the tricarboxylic acid (TCA) cycle, is closely related to the metabolic function of mitochondria [43,44]. Since NAT2 catalyzes the acetyl transfer reaction of acetyl-CoA and plays a regulatory role in maintaining acetyl-CoA homeostasis [45], we further measured the level of acetyl-CoA and other TCA cycle-related metabolites in scleral fibroblasts. The deficiency of NAT2 in scleral fibroblasts led to reduction in the level of acetyl-CoA, acetyl-CoA/CoA ratio, citrate, and α-ketoglutarate (αKG), while the level of lactate, the end product of glycolysis, was increased (Fig. 5F). In contrast, overexpression of NAT2 reversed the above condition (Fig. 5G). The results suggested that in scleral fibroblasts, NAT2 acted as a regulator of acetyl-CoA homeostasis and downstream mitochondrial metabolic pathways. Dysfunction of mitochondria is closely associated with oxidative stress [46], and we found an inhibition effect of NAT2 on mitochondrial ROS generation in scleral fibroblasts (Fig. 5H to J). Overall, the data illustrated that NAT2 mediated mitochondrial function and inhibited oxidative stress in scleral fibroblasts.

### NAT2 regulates phenotypic transition of scleral fibroblasts through ROS/TGF-β pathway

Since several oxidative stress-induced regulatory factors have been reported participating in the phenotypic transition of fibroblasts such as transforming growth factor-β1 (TGF-β1), TGF-β2, connective tissue growth factor (CTGF), and Wnt1 signaling pathway protein 1 (WISP1) [47–49], we wondered whether these regulatory factors were associated with the effect of NAT2 on scleral fibroblasts. Firstly, in vivo and in vitro experiments showed that knockout or knockdown of *Nat2* led to up-regulation of TGF-β1 and TGF-β2 in sclera fibroblasts, but had no significant effects on the level of CTGF or WISP1 (Fig. S3 and Fig. 5K to N). Since TGF-βs could be externally secreted to ECM by fibroblasts and regulated the accumulation of cytokines [50], we extracted the ECM of scleral fibroblasts (Fig. 5O) and found that ECM from fibroblasts with NAT2 knockdown contained higher levels of TGF-βs and cytokines compared with the controls (Fig. 5P). To further identify the relationship of NAT2 and TGF-βs in phenotypic transition of scleral fibroblasts, we used a TGF inhibitor (galunisertib) to treat scleral fibroblasts with NAT2 knockdown and found that it could reverse the phenotypic transition of scleral fibroblasts to myofibroblasts (Fig. 5Q). Thus, TGF-βs participated in the NAT2-regulated phenotypic transition of scleral fibroblasts.

Considering the relationship between NAT2 and ROS generation, we further explored whether NAT2 regulated the level of TGFs in scleral fibroblasts through oxidative stress. In scleral fibroblasts with NAT2 knockdown, both broad-spectrum ROS inhibitor [*N*-acetyl-l-cysteine (NAC)] and mitochondrial-specific ROS inhibitors (Mito-TEMPO) treatment decreased the level of TGF-βs and inhibited phenotypic transition (Fig. S4 and Fig. 5R). Taken together, the results suggest that NAT2 regulates phenotypic transition of scleral fibroblasts through the ROS/TGF-β pathway.

### NAT2-mediated scleral ECM regulates choroidal vascular function

Our findings have suggested the up-regulation of TGF-βs in myopic sclera, especially in ECM, which might have effects on adjacent tissues of sclera. Given that TGF-βs are key regulators of vascular function [51] and choroidal vascular dysfunction is a well-established contributor to myopia pathogenesis [52], we sought to investigate whether ECM derived from NAT2-altered scleral fibroblasts could influence choroidal vascular function. We performed a sprouting assay using murine sclera–choroid complexes. Sub-Tenon’s capsule injection of AAV-sh*Nat2* exacerbated choroidal vascular dysfunction, whereas AAV-*Nat2* administration attenuated it (Fig. S5A). Notably, neither *Nat2* mRNA nor NAT2 protein expression was significantly altered in the choroid of AAV-injected mice (Fig. S5B and C), suggesting that NAT2 in the sclera indirectly modulates choroidal vascular function. To further explore this mechanism, we cultured choroidal microvascular endothelial cells on ECM produced by NAT2-knockdown scleral fibroblasts (Fig. S5D). These cells exhibited significantly enhanced proliferation, migration, and tube formation capabilities, which could be suppressed by the administration of the TGF-β inhibitor galunisertib (Fig. S5E to G). Collectively, these results indicated that NAT2 might mediate crosstalk between scleral ECM and choroidal vascular function in myopia, potentially through the regulation of TGF-β secretion.

### αKG supplementation ameliorated myopia in murine model

**Fig. 6.**
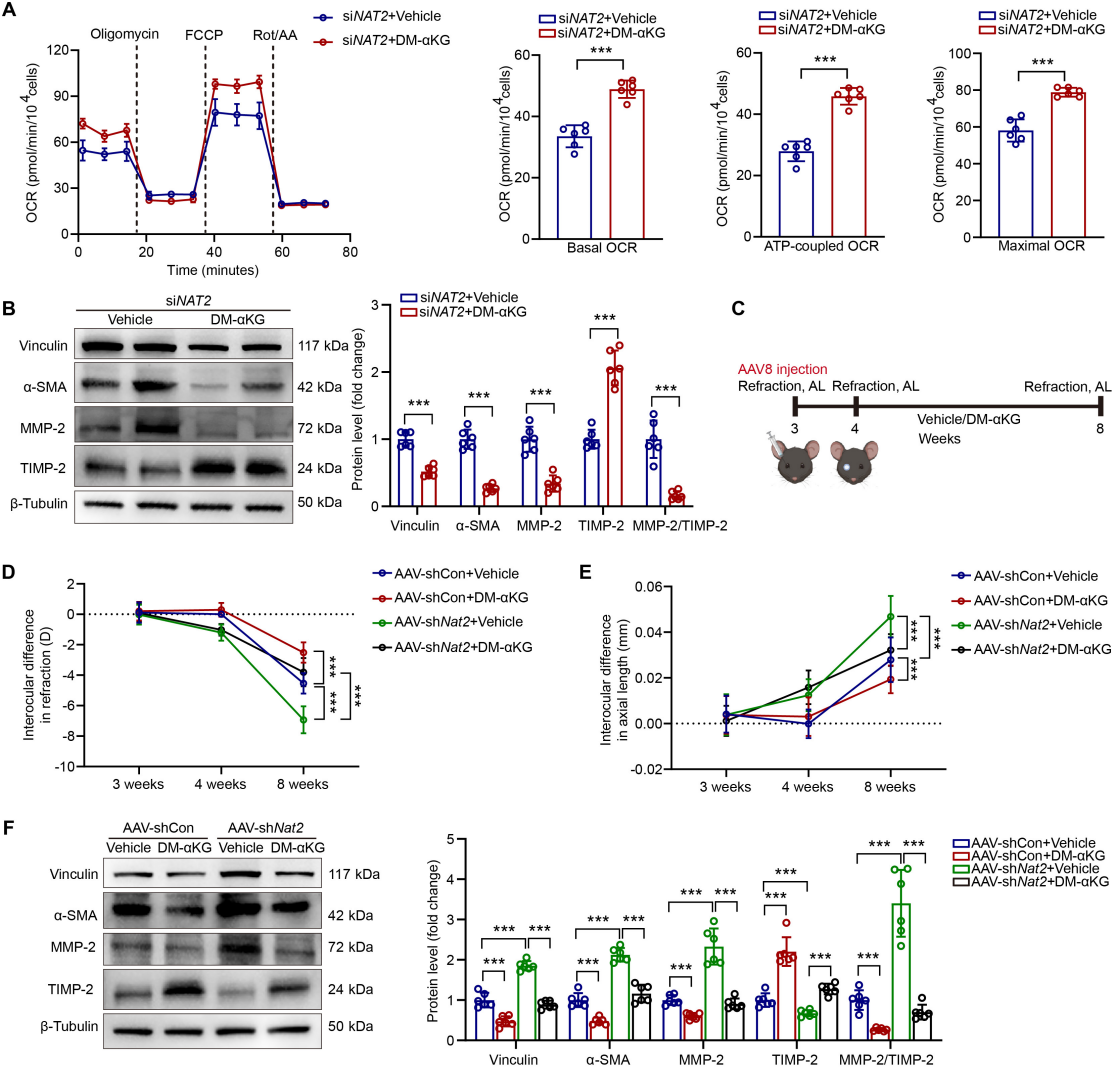
αKG inhibits phenotypic transition of scleral fibroblasts in myopia. (A) Mitochondrial stress test of human scleral fibroblasts with siNAT2 and DM-αKG treatment (*n* = 6 independent experiments). ***P < 0.001. (B) Protein expression of vinculin, α-SMA, MMP-2, and TIMP-2 in human scleral fibroblasts with siNAT2 and DM-αKG treatment measured by Western blot (*n* = 6 independent experiments). ***P < 0.001. (C) Genetic intervention of NAT2, αKG administration, and FDM induction in mice. (D) Interocular difference in refraction of FDM mice with AAV-sh*Nat2* and DM-αKG treatment (*n* = 6 per group). ***P < 0.001. (E) Interocular difference in AL of FDM mice with AAV-sh*Nat2* and DM-αKG treatment (*n* = 6 per group). ***P < 0.001. (F) Protein expression of vinculin, α-SMA, MMP-2, and TIMP-2 in murine sclera of FDM mice with AAV-sh*Nat2* and DM-αKG treatment measured by Western blot (*n* = 6 per group). ***P < 0.001. Data are expressed as mean ± SD.

The above findings have revealed that NAT2 deficiency in scleral fibroblasts contributed to dysfunction of mitochondria as well as the decreases of related metabolites, such as acetyl-CoA, citrate, and αKG. Thus, we hypothesized that rescuing mitochondrial function was considered as potential therapeutic strategies for myopia. Notably, the supplement of αKG is a well-established nutritional supplement in clinic [53] and it has been proved effective in restoring mitochondrial function [53–56]. Thus, we chose αKG for preclinical research attempts. To examine whether αKG administration could influence phenotypic transition of scleral fibroblasts and myopia development, the scleral fibroblasts or mice with FDM were administered vehicle or dimethyl α-ketoglutarate (DM-αKG) [56]. In vitro, we found that DM-αKG significantly promoted mitochondrial metabolism in scleral fibroblasts with NAT2 knockdown (Fig. 6A) and suppressed the expression of myofibroblast markers and MMP-2 as well (Fig. 6B). In FDM mice, αKG treatment suppressed myopia development and reversed the detrimental effect of NAT2 deficiency on myopia (Fig. 6C to E). Consistently, sclera from mice with αKG treatment showed lower levels of myofibroblast markers and MMP-2, compared with those treated with vehicle (Fig. 6F). The findings suggested supplementation of αKG as a potential intervention for myopia.

## Discussion

**Fig. 7.**
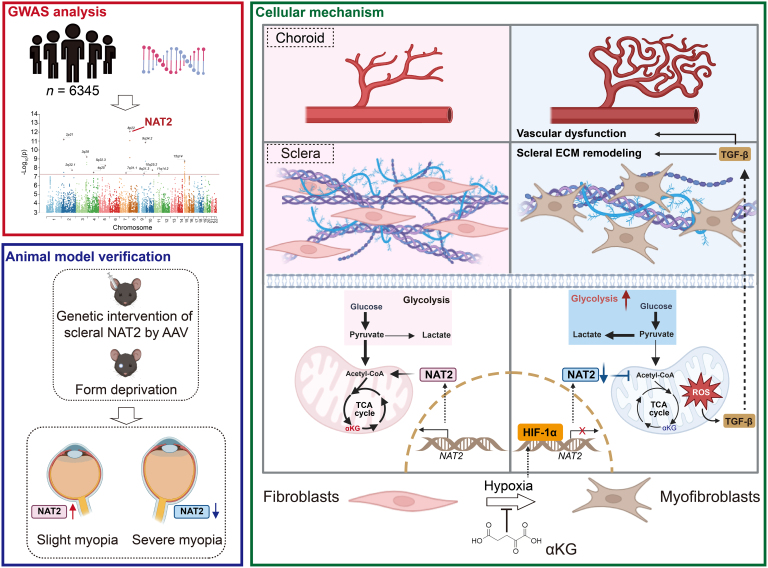
The schematic illustrates the proposed mechanism by which NAT2 protects against myopia. GWAS analysis identified NAT2 as a risk-associated gene. Animal model verification validation confirmed the down-regulation of NAT2 in myopic sclera and demonstrated that genetic modulation of NAT2 expression in murine sclera influenced myopia progression. As for the cellular mechanism, hypoxia-activated HIF-1α suppressed NAT2 expression in scleral fibroblasts, which promoted scleral ECM remodeling and fibroblast phenotypic transition via the mitochondrial ROS/TGF-β pathway. The process also exerted effects on choroidal vascular function through extracellular mechanisms.

This study identified *NAT2* as a risk-associated gene for myopia through GWAS analysis of AL in a youth population. Subsequent experimental validation confirmed the HIF-1α-induced down-regulation of NAT2 in myopic sclera and demonstrated that genetic modulation of NAT2 expression in murine sclera directly influenced myopia progression. Mechanistically, we revealed that NAT2 regulated scleral ECM remodeling and fibroblast phenotypic transition by maintaining mitochondrial metabolism and inhibiting the ROS/TGF-β pathway, which also exerted effects on choroidal vascular function through extracellular mechanisms. Importantly, we identified αKG supplementation as a potential therapeutic strategy, providing a theoretical foundation for novel myopia interventions targeting NAT2 and mitochondrial metabolism (Fig. 7).

To explore potential intervention targets of myopia, GWAS has been extensively employed to identify myopia risk-associated genes [15–19]. However, the selection of dependent variables in GWAS studies on myopia often varies across different investigations. Some studies have focused on the degree of myopia [29,57–59], while others concentrated on the existence of pathological myopia [60,61]. Given that AL elongation represents the most common underlying mechanism of myopia progression and serves as the primary driver of myopic fundus changes, several GWAS have specifically targeted AL to uncover myopia-related genetic factors. For instance, through GWAS, Lu et al. [62] identified 4 novel SNPs related to AL in highly myopic eyes and obtained 2 genes associated with myopia; Han et al. [63] found 2 population-specific loci for AL; and Fuse et al. [64] identified 31 loci associated with AL, 7 of which were newly reported. In the current study, we found the novel AL-related gene *NAT2* and verified its role in regulating myopia progression. These findings highlight the power of GWAS in unraveling the genetic architecture of myopia and provide promising targets for intervention. Moving forward, integrating multi-omics bioinformatics analyses with functional validation will further enhance our ability to decipher the molecular mechanisms of myopia and develop precision medicine strategies for its management.

Our findings established an association between myopia pathogenesis and NAT2, an enzyme involved in mitochondrial metabolism [20,65]. NAT2 has been reported associated with several diseases through its function of metabolism regulation, such as cancer [66,67], neurodegenerative diseases [68,69], and diabetes [21,22]. Growing evidence suggests that myopia is closely linked to metabolic dysregulation in sclera. During myopia progression, the sclera undergoes remarkable structural remodeling, including reduced collagen fibril diameter and ECM dysregulation, which are driven by metabolic alterations [70]. A previous study implicated the role of lipid metabolism regulators in regulating AL elongation through scleral stromal fibroblasts [71]. Further investigations were conducted in the aspect of energy metabolism in sclera fibroblasts. Notably, scleral hypoxia is a critical process in the pathogenesis of myopia [6], which triggers metabolic reprogramming in sclera. Lin et al. [5] demonstrated that glycolytic activation in scleral fibroblasts promoted myopia progression via lactate-induced histone lactylation. Our work shifts focus on another important type of energy metabolism, mitochondrial respiration, revealing its contribution to scleral pathology in myopia. Specifically, we indicate that NAT2 modulates scleral fibroblast phenotype and ECM remodeling by maintaining mitochondrial homeostasis and reducing oxidative stress, thereby influencing MMP-2 activity and inflammatory factor accumulation. These findings emphasize the crucial involvement of mitochondrial metabolism in myopia, with NAT2 emerging as a promising intervention target.

The study advances our understanding of sclera–choroid interactions in myopia. Previous researches have established that choroidal vascular dysfunction contributes to scleral ECM remodeling by reducing oxygen and nutrient supply, leading to scleral hypoxia and subsequent collagen disorganization [6–10]. Here, we further demonstrate that scleral ECM remodeling reciprocally exacerbates choroidal vascular dysfunction. Specifically, we confirmed that regulators like TGF-βs secreted by NAT2-modified scleral fibroblasts directly impaired choroidal endothelial function. This bidirectional interaction suggests a self-perpetuating vicious cycle in myopia progression, underscoring the importance of early intervention.

Notably, the role of TGF-βs in myopia is still not entirely clear. Some studies reported that decreases of TGF-βs were associated with myopia development in animal models, as TGF-βs could promote collagen production, which was critical for the scleral biomechanics [70,72,73]. However, some studies have presented seemingly different viewpoints, which found increases of TGF-βs in myopic eyes and illustrated that TGF-βs could accelerate the progression of myopia through various pathways, like inflammation, apoptosis, and MMP activation [74–77]. In the current study, we found that TGF-βs promoted myopia by targeting scleral fibroblast phenotypic transition and choroidal vascular dysfunction. The differences in these research may stem from the variations among different myopic animal models, and the changes of TGF-βs during the progression of myopia are dynamic, with both temporal and spatial characteristics [76]. Future studies describing the distribution of TGF-βs in different tissues of myopic eyes will further clarify the role of TGF-βs in myopia.

We made a preliminary attempt to use αKG to treat myopia, which provided a potential strategy in myopia management. Although previous studies have explored several novel methods to control myopia, such as low-concentration atropine [78], low-level red light [79], and violet light [80], the effective etiological treatments remain limited. Additionally, dietary modification has emerged as a myopia control strategy. Evidence suggests that limiting sugar intake [5] and increasing dietary ω-3 polyunsaturated fatty acids [81] may help protect against myopia. In the current study, we verified the effect of αKG on myopia, which is a downstream metabolite of NAT2 and a common dietary nutritional supplement. The supplementation of αKG has been shown to benefit various diseases through its role in maintaining mitochondrial metabolism and cellular energy homeostasis [56,82,83]. Importantly, our findings suggest that αKG may mitigate myopia progression by suppressing scleral ECM remodeling and fibroblast phenotypic transition. Given its safety profile and broad biological functions, αKG represents a promising nutraceutical intervention for myopia control.

Although this study has made important progress in revealing the role of NAT2 in myopia, certain limitations should be acknowledged. First, while our experimental models provide robust mechanistic insights, the translational relevance of these findings in human myopia requires further validation through clinical studies. Second, we used AAV to achieve NAT2 knockdown and overexpression in murine scleral fibroblasts. Although AAV is widely used for gene manipulation, its long-term safety profile, such as the potential risk of inducing scleral inflammatory responses, remains to be thoroughly evaluated. Future studies employing tissue-specific knockout models would not only strengthen the causal inferences but also help circumvent possible off-target or immunogenic effects associated with viral vectors. Moreover, the current study focuses primarily on scleral fibroblasts; however, NAT2 is also expressed in other ocular tissues, such as the retina, which plays a key role in visual signal modulation and myopic progression. Whether NAT2 expression in these tissues contributes to myopia pathogenesis warrants further investigation. Additionally, given that myopia is a polygenic disorder, it is essential to explore potential genetic interactions between NAT2 and other established myopia-associated genes. Such studies could provide a more comprehensive understanding of the genetic network underlying myopia and clarify whether NAT2 acts independently or synergistically within these pathways.

In conclusion, *NAT2* represents a newly identified risk-associated gene of myopia, serving as a key regulator of disease progression. Through the mitochondrial ROS/TGF-β pathway, NAT2 modulates scleral ECM remodeling and fibroblast phenotypic transition, with downstream effects on choroidal vascular function. Both genetic manipulation of NAT2 and αKG supplementation demonstrate therapeutic potential in experimental myopia, offering novel strategies for disease prevention and treatment. These findings not only provide new insights into myopia pathogenesis but also pave the way for innovative interventions targeting metabolic pathways in ocular tissues.

## Materials and Methods

### Human studies

For the human study, we utilized data from a previously established cohort in the Shanghai Time Outside to Reduce Myopia (STORM) trial (ClinicalTrials.gov Identifier: NCT02980445) [84]. Prior to participation, the study protocol was thoroughly explained to all participants, and written informed consent was obtained from their parents or legal guardians. This study was conducted according to the principles of the Declaration of Helsinki and received approval from the Institutional Review Board of Shanghai General Hospital, Shanghai Jiao Tong University. Biometric data were obtained from school records. Each participant accepted a series of ophthalmic examinations, including cycloplegic refraction and AL measurement. Genomic DNA was isolated from peripheral blood samples of participants, and genome-wide association analysis for AL was conducted. Details of obtaining human data and GWAS analysis were shown in the Supplementary Materials.

### Animal studies

All procedures adhered to the National Institutes of Health Guidelines for the Care and Use of Laboratory Animals (8th edition, 2011) and were approved by the Animal Ethics Committee of Fudan University (20230301-118). FDM was induced in C57BL/6 male mice by affixing homemade translucent occluders to the right eye, secured with plastic collars to prevent displacement [85]. To explore the effects of NAT2 on scleral remodeling during myopia development, we employed both viral-mediated gene manipulation and genetic knockout approaches. For gene modulation studies, we generated AAV8 vectors encoding either mouse *Nat2* short hairpin RNA (shRNA) (AAV-sh*Nat2*) or full-length *Nat2* (AAV-*Nat2*), along with their respective controls (AAV-shCon and AAV-Con). Three-week-old wild-type C57BL/6J mice received unilateral sub-Tenon’s capsule injections of 5-μl viral suspension in the right eye, following established protocols [5]. In parallel, we generated *Nat2* knockout mice (*Nat2*^−*/*−^) through CRISPR/Cas9-mediated gene editing to further examine the consequences of NAT2 deficiency on ocular development and myopia susceptibility. The details of the animal studies were shown in the Supplementary Materials.

### Cell studies

Primary human scleral fibroblasts were obtained from human sclera tissue (Mcellbank, China) and cultured in specific culture medium for fibroblasts (M-CH-1201-500; Mcellbank, China) in a 5% CO_2_ incubator, at 37 °C. Human choroidal microvascular endothelial cells were obtained from human choroid tissue (Mcellbank, China) and cultured in Endothelial Cell Medium (1001; Sciencell, USA) in a 5% CO_2_ incubator at 37 °C.

### Statistical analysis

Quantitative data are presented as means with SDs; qualitative data are presented as frequencies (percentages). Normality of data distribution was assessed using the Shapiro–Wilk test, and the equality of variances was determined using the Levene test. For 2-group comparisons, normally distributed variables were analyzed using Student’s *t* test (equal variances) or unequal variance *t* test (unequal variances), with nonparametric Mann–Whitney *U* tests applied otherwise. Multi-group comparisons utilized 2-way analysis of variance (ANOVA) with Bonferroni post hoc analysis. Categorical variables were compared using chi-square or Fisher’s exact tests. Analyses were conducted using SPSS 25.0 and GraphPad Prism 8, with statistical significance set at *P* < 0.05.

Experimental details are presented in the Supplementary Materials.

## Data Availability

The data supporting the findings of this study are available within the manuscript and its Supplemental Materials. All other data are available from the corresponding author upon reasonable request (xianhezi@163.com).

## Supplementary Materials

Supplementary 1 Experimental Details Figs. S1 to S5 Tables S1 and S2 References [86,87]
