# Supplementary material for: N-Acetyltransferase 2 Inhibits Myopia by Maintaining Mitochondrial Metabolism in Scleral Fibroblasts
Source: Research (Wash D C). 2025 Oct 29;8:0969. doi: 10.34133/research.0969 (PMC12569602; doi:10.34133/research.0969)
Supplement: Supplementary 1 — Experimental Details Figs. S1 to S5 Tables S1 and S2 References [86,87] [file research.0969.f1.docx]

**Supplementary materials**

**Experimental details**

**Tables S1-S2**

**Figures S1-S5**

**Uncropped gels of Western blot**

**Experimental Details**

**Study population**

For the current analysis, we utilized data from a previously established cohort in the Shanghai Time Outside to Reduce Myopia (STORM) trial (ClinicalTrials.gov Identifier: NCT02980445) [84]. The STORM trial enrolled children and adolescents (4-18 yrs) from schools in Shanghai. Written informed consent was obtained from parents or legal guardians of all participants prior to enrollment, and the study protocol was approved by the Institutional Review Board of Shanghai General Hospital, Shanghai Jiao Tong University, in accordance with the principles of the Declaration of Helsinki. Participants with organic ocular diseases except myopia (amblyopia, strabismus, congenital cataract and glaucoma), prior myopia control treatment, systemic conditions, or those who did not undergo cycloplegia were excluded from the study. After applying these criteria, a total of 6345 participants were included in the final analysis.

**Clinical and laboratory assessments**

Cycloplegia was conducted by administering a single drop of 0.5% proparacaine hydrochloride (topical anesthesia) in each eye, followed by two drops of 1% cyclopentolate (Cyclogyl; Alcon, USA) with 5-minute intervals. After 40 minutes, if the eyes were with pupillary dilation ( > 6 mm) and absence of light reflex, cycloplegia was considered successful; otherwise, an additional drop of cyclopentolate was administered to the eye. Intraocular pressure was measured by a non-contact tonometer (NT-1000; Nidek, Japan). Cycloplegic refraction was performed with an autorefractor (KR-8900; Topcon, Japan). Three consecutive measurements were taken; if any two readings differed by > 0.50 D, the measurements were repeated. AL was measured by an IOL Master (Carl Zeiss Meditec, Germany). Three consecutive measurements were taken; if any two readings differed by > 0.05 mm, the measurements were repeated.

**Genomic DNA extraction and GWAS analysis**

Genomic DNA was isolated from peripheral blood samples using FastPure Blood DNA Isolation Mini Kit (DC112; Vazyme, China). DNA quality was assessed based on the following criteria: concentration (25–150 ng/μL), purity (OD260/280: 1.6–2.1), and total content (≥ 500 ng).

Genotyping was performed using the Infinium Asian Screening Array-24 v1.0 BeadChip. Raw data were processed with GenomeStudio v2.0. The quality control, phasing and imputation of data were conducted using the Rapid Imputation and Computational Pipeline for GWAS. Phasing was performed with Eagle, and variants were imputed against the 1000 Genomes Project Phase 3 reference panel (Minimac). Retained variants met the following criteria: imputation quality (info ≥ 0.4) and minor allele frequency (MAF ≥ 0.01). Genome-wide association analysis for AL employed linear regression adjusted for age, sex, and the top five principal components.

**Animal studies**

All procedures adhered to the National Institutes of Health Guidelines for the Care and Use of Laboratory Animals (8th edition, 2011) and were approved by the Animal Ethics Committee of Fudan University (20230301-118). Mice were housed under a 12-h light/dark cycle at 24 ± 2°C and 40 ± 5% humidity with ad libitum access to food and water.

FDM was induced in four-week-old C57BL/6 male mice by affixing homemade translucent occluders to the right eye, secured with plastic collars to prevent displacement[85]. Refractive error was measured using an eccentric infrared photorefractor specifically configured for the murine eye in a dark environment[85]. Each animal was gently restrained without anesthesia, and its position was adjusted according to the existence of the first clear Purkinje image at the center of the pupil. Refractive measurements were repeated three times, and the mean was taken as the final value. AL was measured using a custom ultra-long depth SD-OCT system[86]. After anesthesia (0.6% pentobarbital), mice were positioned on a stage equipped with a modified slit lamp and scanned along the optical axis via an X-Y cross-scanning system. Custom software was used to process raw OCT data and extract axial ocular dimensions. AL measurements were repeated three times, and the mean was taken as the final value. To explore the effects of NAT2 on scleral remodeling during myopia development, we employed both viral-mediated gene manipulation and genetic knockout approaches. For gene modulation studies, we generated AAV8 vectors encoding either mouse *Nat2* shRNA (AAV-sh*Nat2*) or full-length *Nat2* (AAV-*Nat2*), along with their respective controls (AAV-shCon and AAV-Con), with titers ranging from 1.0×10¹³ to 1.5×10¹³ genomic copies/mL. Three-week-old wild-type C57BL/6J mice received unilateral sub-Tenon's capsule injections of 5 μL viral suspension in the right eye, following established protocols[5]. Post-operative care included application of 0.2% polyacrylic acid ocular dressing for 3 days to prevent infection and promote healing. In parallel, we generated *Nat2^⁻/⁻^* mice through CRISPR/Cas9-mediated gene editing to further examine the consequences of NAT2 deficiency on ocular development and myopia susceptibility.

To confirm the effects of dimethyl α-ketoglutarate (DM-αKG) (349631, Sigma, MO, USA) on myopia, FDM mice were intraperitoneally injected with DM-αKG (100 mg/kg/day) or vehicle for 28 days. DM-αKG was dissolved in dimethyl sulfoxide (DMSO) and further diluted in saline.

**Cell culture**

Primary human scleral fibroblasts were obtained from human sclera tissue (Mcellbank, China) and cultured in specific culture medium for fibroblasts (M-CH-1201-500; Mcellbank, China) in a 5% CO_2_ incubator, at 37 ℃. The condition of fibroblasts was assessed before experiments through morphology observation and immunofluorescence staining of vimentin. The fibroblasts that have undergone no more than seven passages would be used. Human choroidal microvascular endothelial cells were obtained from human choroid tissue (Mcellbank, China) and cultured in Endothelial Cell Medium (1001; Sciencell, USA) in a 5% CO_2_ incubator, at 37 ℃. The condition of endothelial cells was assessed before experiments through morphology observation and immunofluorescence staining of CD31. The endothelial cells that have undergone no more than seven passages would be used.

**Immunofluorescence staining**

Freshly eyeballs were rinsed with saline and immediately embedded in optimal cutting temperature (OCT) compound (4583; SAKURA Tissue-TEK, USA). Cryosections (5 μm thickness) were prepared and incubated overnight at 4°C with primary antibodies against NAT2 (1:1000, 11410-1-AP; Proteintech, USA) and Vimentin (1:1000, 60330-1-Ig; Proteintech, USA). Sections were then incubated with Alexa Fluor 555- and 488-conjugated secondary antibodies (1:200, Invitrogen, USA) at 37°C in the dark for one hour. Nuclei were counterstained with 4′,6-diamidino-2-phenylindol (DAPI) (P36931; Invitrogen, USA), and images were acquired by a confocal microscope (FV3000; Olympus, Japan).

**Protein extraction and Western blot**

Total proteins were extracted from cells and ocular tissues with RIPA lysis buffer. Protein samples (20-30 μg) were separated by 12.5% SDS-PAGE and transferred to nitrocellulose membranes. After blocking with blocking buffer (PS108P; Epizyme, China), membranes were incubated overnight at 4°C with primary antibodies against NAT2 (1:1000, 11410-1-AP; Proteintech, USA), β-Tubulin (1:1000, 10068-1-AP; Proteintech, USA), Vinculin (1:1000, 66305-1-Ig; Proteintech, USA), α-SMA (1:1000, ab7817; Abcam, USA), MMP-2 (1:1000, 10373-2-AP; Proteintech, USA), TIMP-2 (1:1000, 17353-1-AP; Proteintech, USA), TGF-β1 (1:1000, 21898-1-AP; Proteintech, USA), TGF-β2 (1:1000, 19999-1-AP; Proteintech, USA), CTGF (1:1000, 25474-1-AP; Proteintech, USA), WISP1 (1:1000, 18166-1-AP; Proteintech, USA). After the incubation of corresponding secondary antibodies (1:10000, Jackson Immuno Research Laboratories, USA), enhanced chemiluminescence (Share-bio, China) was used to show the protein bands with the ImageQuant LAS 4000 mini detection system (General Electric Co., USA).

**RNA isolation, real-time quantitative PCR (RT-qPCR) and RNA sequencing**

TRIzol was applied to isolate total RNA and cDNA synthesis was conducted with a PrimeScript RT reagent kit (Takara, Japan). The mRNA expression was detected by RT-qPCR with SYBR Premix Ex Taq™ (Takara, Japan) using a LightCycler® 480 Realtime PCR System (Roche Applied Science, Switzerland). *β-tubulin* was used as a housekeeping gene. The primers were shown in Table S2.

For RNA-sequencing, the sclera of FDM mice were extracted rapidly under ice bath, followed by DEPC-treated water washing. The tissues were snap-frozen in liquid nitrogen and then used to isolate total RNA and construct a cDNA library. Then, RNA-sequencing was conducted with high-throughput sequencing service (HaploX, China). Differentially expressed genes were defined as adjusted (Benjamini and Hochberg) *p* < 0.05 and |log_2_ (fold change)| > 1.

**RNA interference and plasmid construction**

The siRNAs targeting human *NAT2,* and shRNA targeting mouse *Nat2* were synthesized by Genepharma Co. Ltd (China) (Table S4). Transfection of siRNAs or plasmid was performed using lipofectamine 3000 reagent (L3000015; Thermo-Fisher, USA). The interfering efficiency was validated by RT-qPCR and Western blot.

**Chromatin Immunoprecipitation (ChIP)**

ChIP assays were performed with a SimpleChIP® Plus Enzymatic Chromatin IP Kit (9005; Cell Signaling Technology, USA). In brief, scleral fibroblasts were cultured under normoxia or hypoxia for 24 h and then cross-linked with 1% formaldehyde. Then, the extracted chromatin was digested by micrococcal nuclease, followed by sonication for lysis. ChIP was conducted with antibodies against HIF-1α (20960-1-AP; Proteintech, USA), Histone H3 (17168-1-AP; Proteintech, USA), and normal rabbit IgGs (2729; Cell Signaling Technology, USA). After DNA purification, immunoprecipitated DNA was analyzed by RT-qPCR. Primer information for ChIP is presented in Table S2.

**Dual luciferase reporter assay**

To examine the function of SNP rs78797078, SNP rs78797078 (G allele / A allele) (±200 bp) were subcloned into pGL3-SV40 luciferase reporter vectors to construct pGL3-SNP rs78797078 (G allele / A allele) (±200 bp)-SV40-Luci. Scleral fibroblasts were seeded in a 6-well plate and co-transfected with 1 µg of pRL-TK control vector (Renilla), and 1 µg pGL3-SNP rs78797078 (G allele / A allele) (±200 bp) plasmid (Firefly). Cell lysates were collected at 48 h after transfection.

The binding locus site of HIF-1α on the *NAT2* promoter was predicted (ACGTG) using the online tool JASPAR (http://jaspar.genereg.net/). Then, the wild type human *NAT2* promoter and a mutated *NAT2* promoter with the deletion of the binding locus site were subcloned into pGL3-basic luciferase reporter vectors to construct pGL3-wt*NAT2*-promoter-Luci and pGL3-mut*NAT2*-promoter-Luci, respectively. Scleral fibroblasts were seeded in a 6-well plate and co-transfected with 1 µg of pRL-TK control vector (Renilla), and 1 µg pGL3-wt*NAT2* plasmid or pGL3-mut*NAT2* plasmid (Firefly). Cell lysates were collected at 48 h after transfection and culture under normoxia or hypoxia.

Luciferase activities were measured by the Dual Luciferase Reporter Gene Assay Kit (RG027; Beyotime, China) using a MultiSkan FC reader (ThermoFisher, USA).

**ECM assays**

ECM extraction was based on a previous study[87]. The culture plates were coated with 1% gelatin and 0.01% collagen I (A1048301; Gibco, USA) for one hour at 37ºC. Then, the gelatin was cross-linked with 1% glutaraldehyde and incubated for 30 minutes at 37ºC. After being washed with PBS and DMEM, the human scleral fibroblasts were seeded and cultured for 5 days to produce ECM. The decellularization was conducted with ice-cold extraction buffer (0.2% of Triton X-100 in ultrapure water) until the fibroblasts were completely lysed (5–10 minutes). Finally, human choroidal microvascular endothelial cells were seeded in the extracted ECM.

**Enzyme-linked immunosorbent assays (ELISAs)**

According to the manufacturer’s instructions, murine sclera, scleral fibroblasts or scleral ECM was homogenized with PBS and centrifuged (4°C, 15,000 × g, 15 min). Then, the supernatants were used to measure TGF-β1, TGF-β2 and cytokines/chemokines (IL-6 and TNF-α) by ELISA kits (MultiSciences, China).

**Measurement of the oxygen consumption rate (OCR)**

Oxygen consumption was measured in a Seahorse XF96 analyzer (Agilent, USA). In brief, human scleral fibroblasts (10,000-20,000 cells/80 µl/well) were seeded in the XF96-well microplate (102601-100; Agilent, USA). Injection ports were loaded with 10× injection mixes to obtain a final concentration of 1.5 µM oligomycin, 2.0 µM FCCP and 0.5 µM rotenone/antimycin after injection using an XF Cell Mito Stress Test Kit (103015–100; Agilent, USA). OCR values were normalized to the number of cells in each well.

**Statistical analysis**

Quantitative data are presented as means with standard deviations; qualitative data as frequencies (percentages). Normality of data distribution was assessed using the Shapiro-Wilk test and the equality of variances was determined using the Levene test. For two groups comparisons, the Student’s *t* test (equal variances) or unequal variance t test (unequal variances) was applied for normally distributed variables, otherwise the Mann-Whitney *U* test was performed. Categorical variables were compared using Chi-square or Fisher's exact tests. Analyses were conducted using SPSS 25.0 and GraphPad Prism 8, with statistical significance set at *p* < 0.05.

**Table S1.** **Characteristics of population**

|  | **Mean** | **SD** | **Range** |
| --- | --- | --- | --- |
| **Age, yrs** | 9.59 | 1.61 | 4~18 |
| **SE, D** | -0.65 | 2.86 | -18.63~8.38 |
| **AL, mm** | 23.73 | 1.29 | 20.16~31.06 |
| **CR, mm** | 7.84 | 0.26 | 6.12~9.12 |

AL: axial length; CR: corneal radius of curvature, SE: spherical equivalent.

**Table S2. Information of PCR primers**

| Gene | Primer | Sequence (5’-3’) | Application |
| --- | --- | --- | --- |
| Nat2 (mouse) | Forward | GTCCTGGTAGCGGGAAACC | RT-qPCR |
|  | Reverse | GGTGCTGAAGGATTTCGGTTAAT | RT-qPCR |
| *β-Tubulin* (mouse) | Forward | TTTTCGTCTCTAGCCGCGTG | RT-qPCR |
|  | Reverse | GATGACCTCCCAGAACTTGGC | RT-qPCR |
| *NAT2* (human) | Forward | TGCTGGCCAAAGGGATCAT | RT-qPCR |
|  | Reverse | CTGGTGCTCAAGAATGTCAGTT | RT-qPCR |
| *β-Tubulin* (human) | Forward | ATCTTTGGTCAGAGTGGGGC | RT-qPCR |
|  | Reverse | CAGGCAGTCGCAGTTTTCAC | RT-qPCR |
| siNAT2-1 (human) |  | UGUUUGUAAUAUACUGCUC tt | siRNA |
| siNAT2-2 (human) |  | UGUAUUUGUUAACUGGAGG tt | siRNA |
| siNAT2-3 (human) |  | UAGUAAGGGAUCCAUCACC tt | siRNA |
| shNat2 (mouse) |  | ACGTCGATCTTGTAGAGTTTA | shRNA |
| *NAT2* promoter (human) | Forward | CTGATCTGCAACTCCACGGT | ChIP PCR |
|  | Reverse | CTGATCTGCAACTCCACGGT | ChIP PCR |


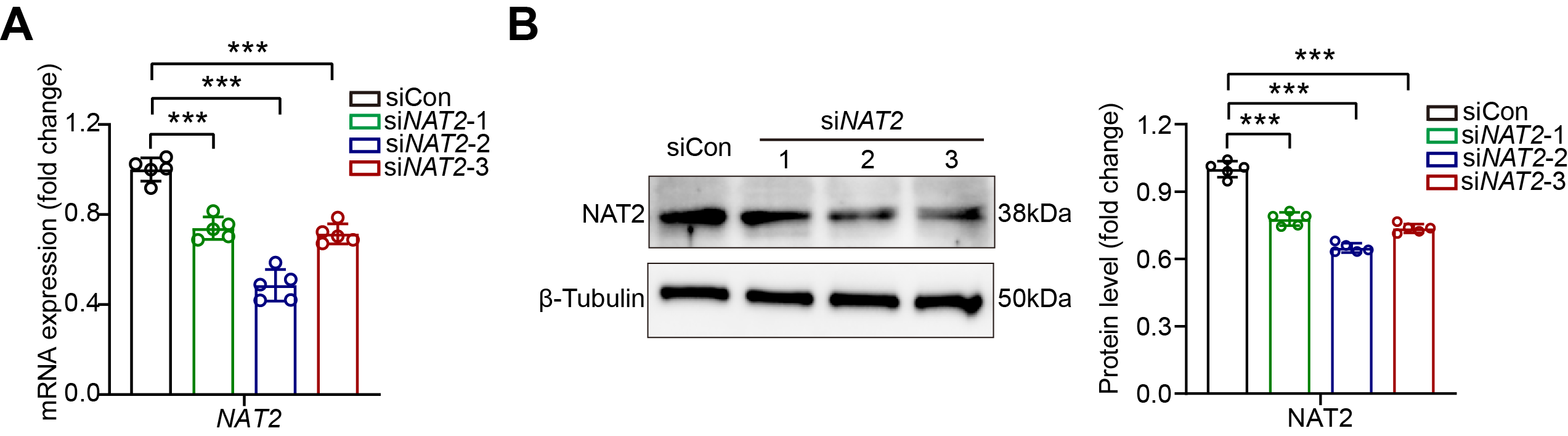


**Figure S1. Knockdown of NAT2 in scleral fibroblasts by siRNA.**

**A**, The mRNA expression of *NAT2* in human scleral fibroblasts transfected with si*NAT2*-1 to -3 (n = 6 independent experiments). ****p* < 0.001. **B**, The protein expression of NAT2 in human scleral fibroblasts transfected with si*NAT2*-1 to -3 (n = 6 independent experiments). ****p* < 0.001. Data are expressed as mean ± SD. NAT2: N-acetyltransferase 2.


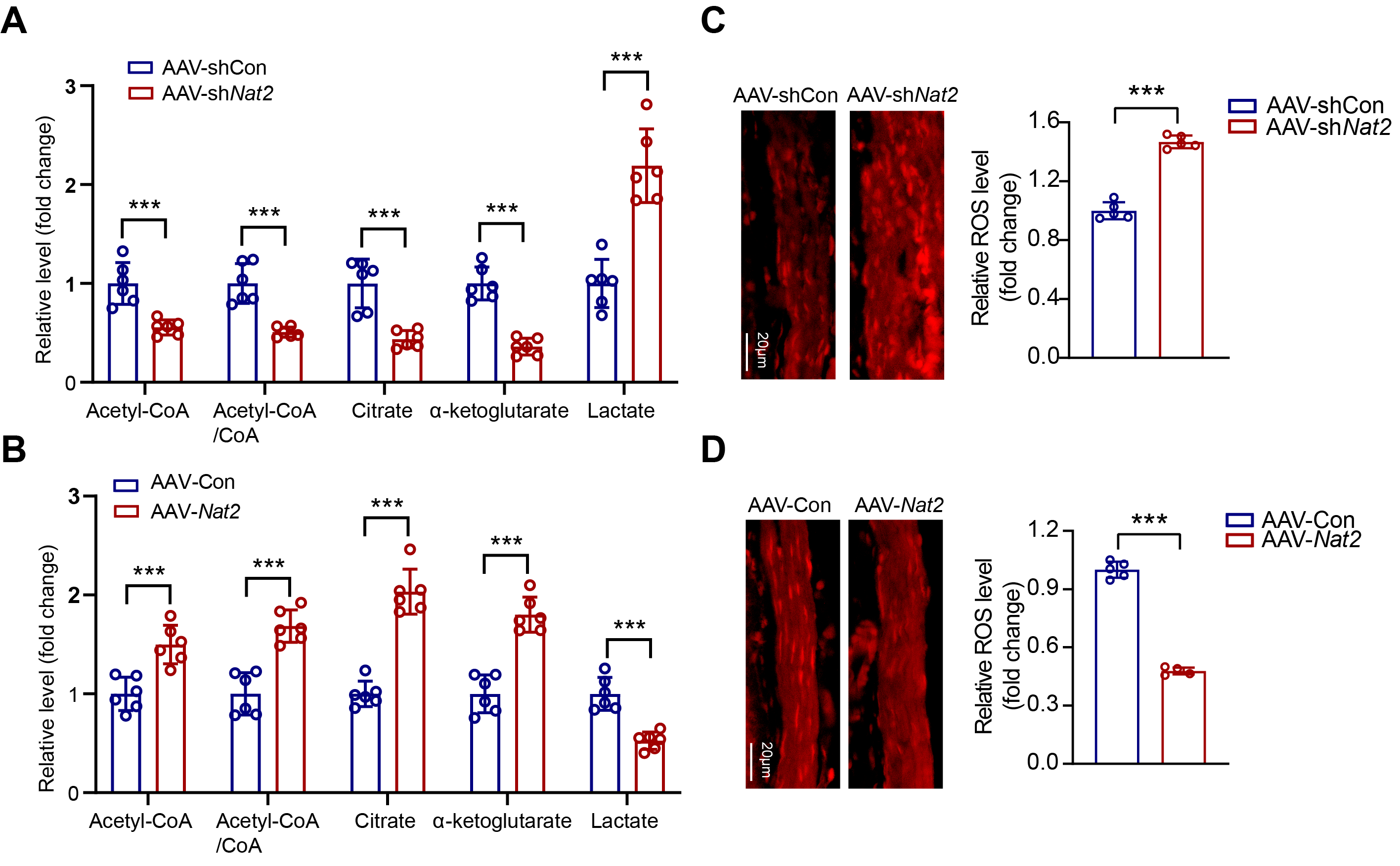


**Figure S2. Genetic intervention of NAT2 changes levels of metabolites and ROS in murine sclera.**

**A&B**, Levels of metabolites in murine sclera of FDM mice with *Nat2* knockdown or overexpression (n = 6 per group). ****p* < 0.001. **C&D**, Level of ROS in murine sclera of FDM mice with *Nat2* knockdown or overexpression (n = 6 per group). ****p* < 0.001. Data are expressed as mean ± SD. AAV: adeno-associated virus; NAT2: N-acetyltransferase 2; ROS, reactive oxygen species.


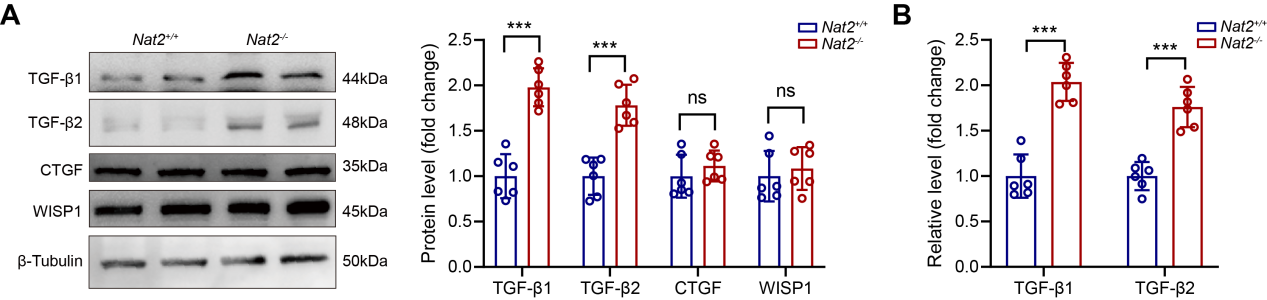


**Figure S3. *Nat2* knockout promotes TGF-βs in murine sclera.**

**A**, Protein expression of TGF-β1, TGF-β2, CTGF and WISP1 in sclera of FDM mice (*Nat2^+/+^* or *Nat2^-/-^*) measured by Western blot (n = 6 per group). ****p* < 0.001; ns, *p* ≥ 0.05. **B**, Levels of TGF-β1 and TGF-β2 in sclera of FDM mice (*Nat2^+/+^* or *Nat2^-/-^*) measured by ELISA (n = 6 per group). ****p* < 0.001. Data are expressed as mean ± SD. CTGF: connective tissue growth factor; ECM: extracellular matrix; ELISA: enzyme-linked immunosorbent assay; NAT2: N-acetyltransferase 2; TGF: transforming growth factor; WISP1: Wnt1 signaling pathway protein 1. AAV: adeno-associated virus; DHE: dihydroethidium; NAT2: N-acetyltransferase 2; ROS: reactive oxygen species.


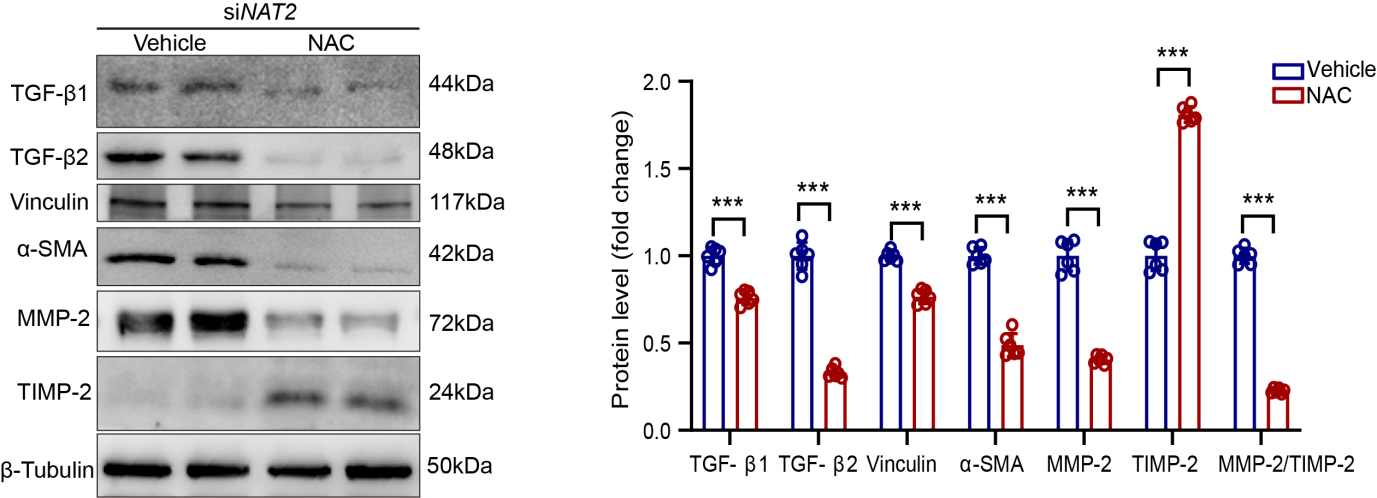


**Figure S4. NAC treatment suppresses phenotypic transition of scleral fibroblasts with NAT2 knockdown.**

Protein expression of TGF-β1, TGF-β2, Vinculin, α-SMA, MMP-2 and TIMP-2 in human scleral fibroblasts with siNAT2 and NAC treatment measured by Western blot (n = 6 independent experiments). ****p* < 0.001. Data are expressed as mean ± SD. α-SMA: α-smooth muscle actin; MMP-2: matrix metallopeptidase 2; NAC: N-acetyl-L-cysteine; NAT2: N-acetyltransferase 2; TGF: transforming growth factor; TIMP-2: tissue inhibitors of metalloproteinase 2; TNF-α: tumor necrosis factor; WISP1: Wnt1 signaling pathway protein 1.

**
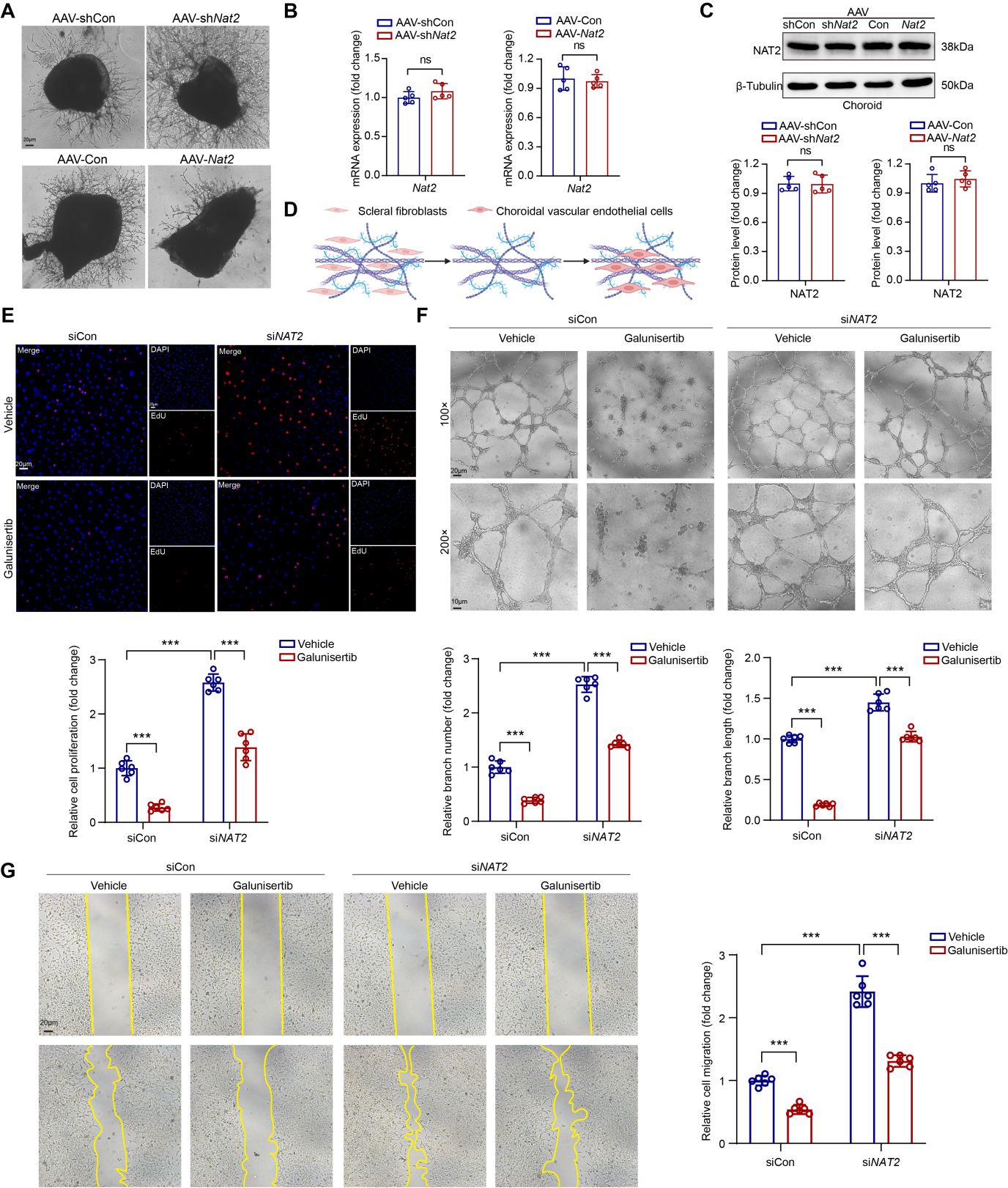
**

**Figure S5. NAT2-regulated scleral ECM affects choroidal vascular function.**

**A**, Choroid sprouting assay of murine eyes with scleral *Nat2* knockdown or overexpression. **B**, Quantification of *Nat2* mRNA expression in choroid of murine eyes with scleral *Nat2* knockdown or overexpression measured by RT-qPCR (n = 6 per group). ****p* < 0.001. **C**, NAT2 protein expression in choroid of murine eyes with scleral *Nat2* knockdown or overexpression measured by Western blot (n = 6 per group). ****p* < 0.001. **D**, Culture of human choroidal microvascular endothelial cells on ECM extracted from human scleral fibroblasts. **E**, TUNEL assay of choroidal microvascular endothelial cells cultured on ECM of human scleral fibroblasts with si*NAT2* and galunisertib treatment (n = 6 independent experiments). ****p* < 0.001. **F**, Tube formation assay of choroidal microvascular endothelial cells cultured on ECM of human scleral fibroblasts with si*NAT2* and galunisertib treatment (n = 6 independent experiments). ****p* < 0.001. **G**, Wound healing assay of choroidal microvascular endothelial cells cultured on ECM of human scleral fibroblasts with si*NAT2* and galunisertib treatment (n = 6 independent experiments). ****p* < 0.001. Data are expressed as mean ± SD. AAV: adeno-associated virus; ECM: extracellular matrix; NAT2: N-acetyltransferase 2; TUNEL, TdT-mediated dUTP Nick-End Labeling.

**Uncropped gels of Western blot**

**
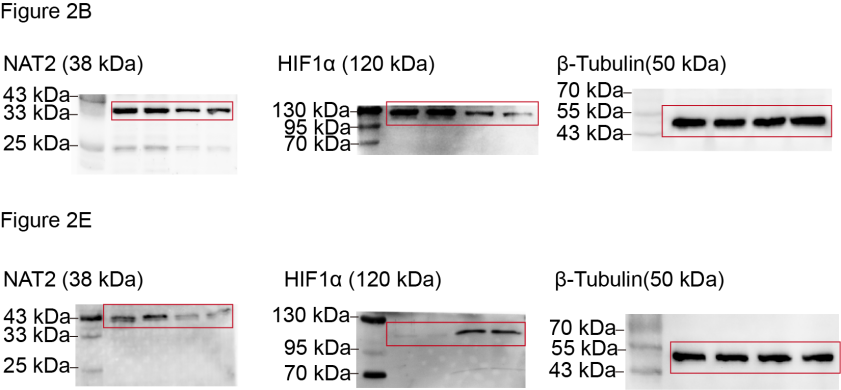
**

**
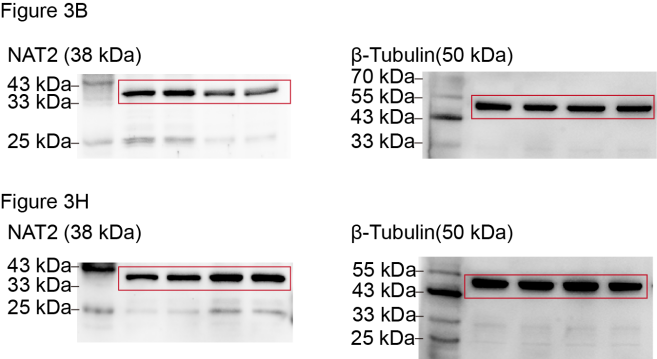
**

**
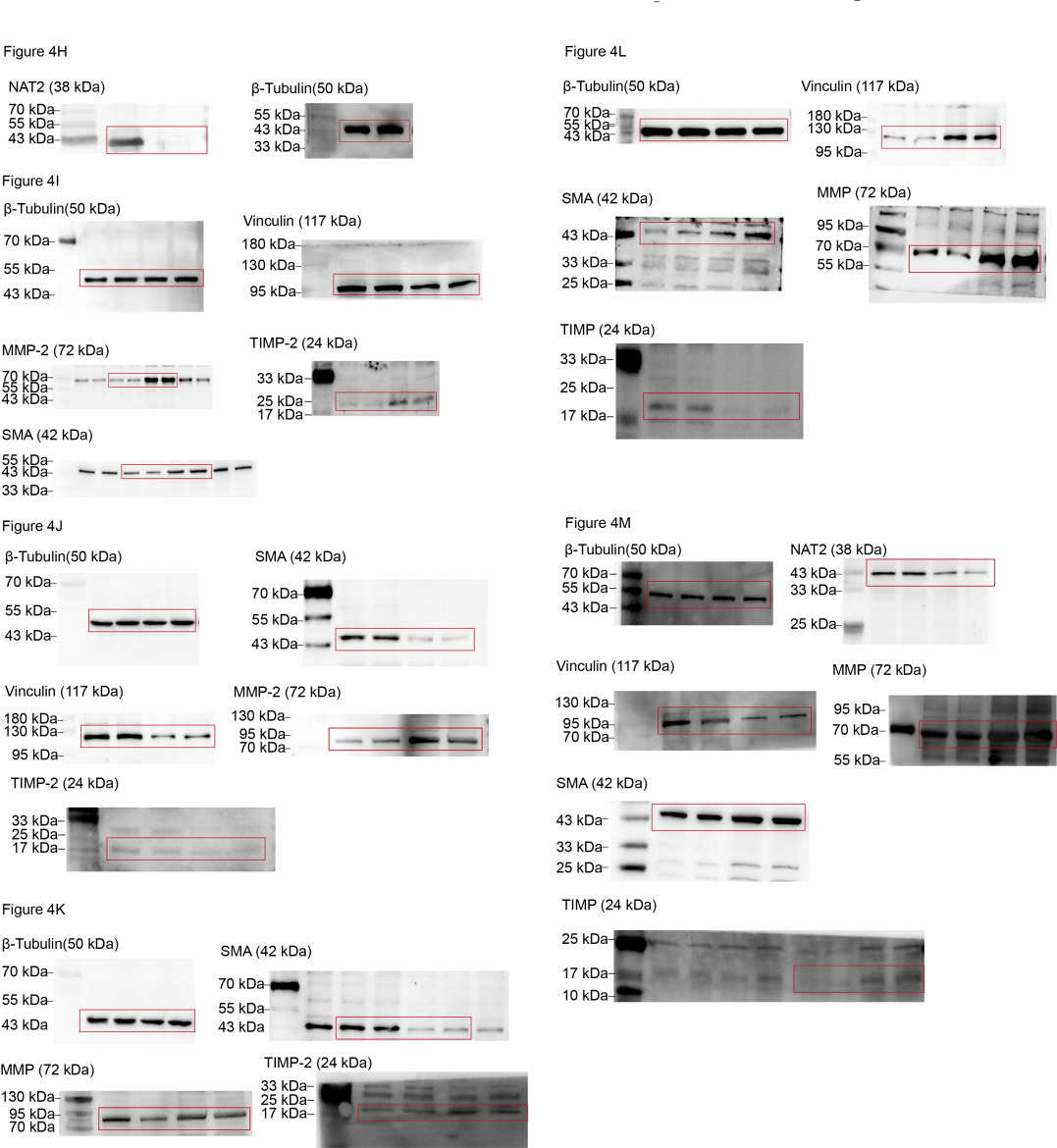
**

**
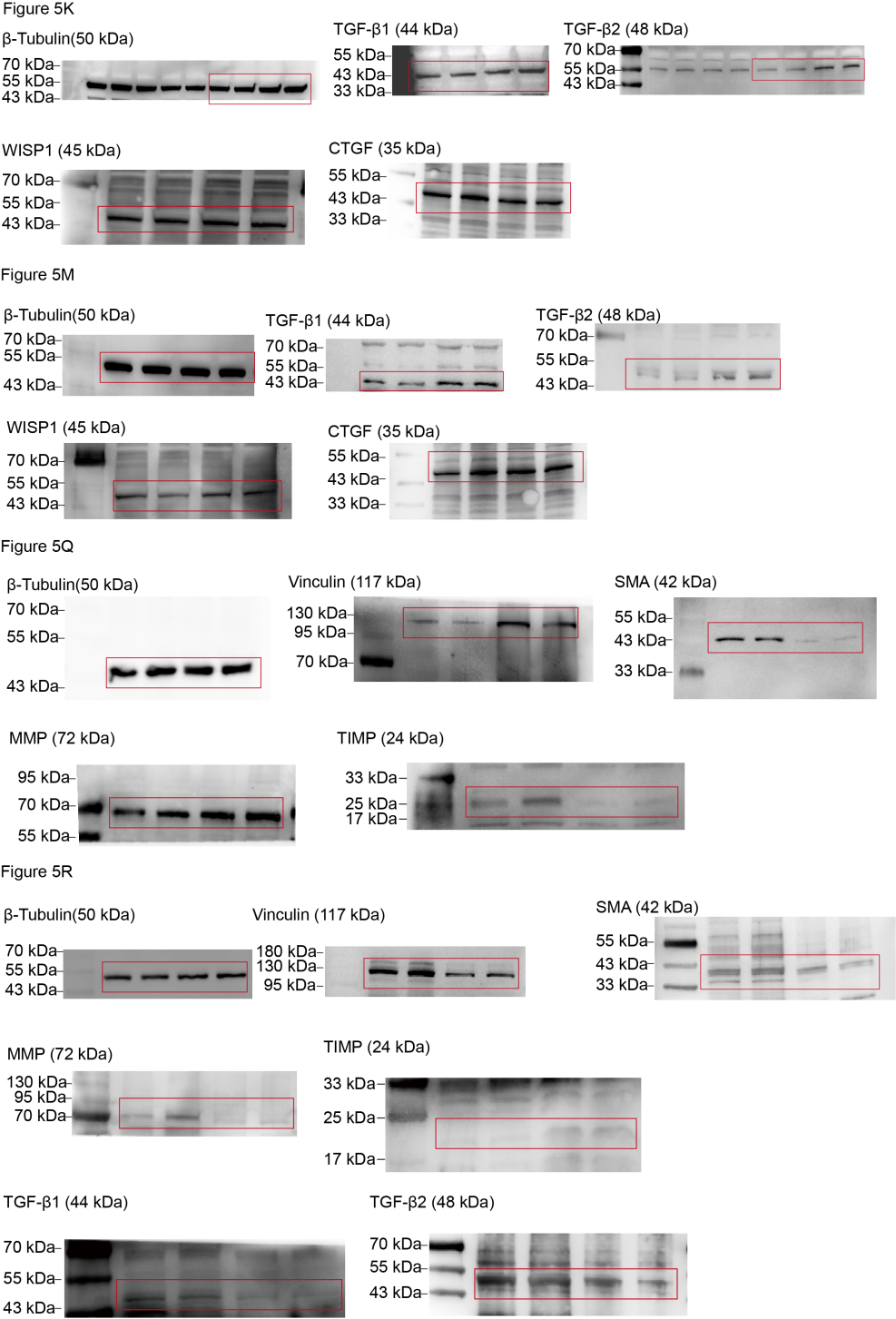

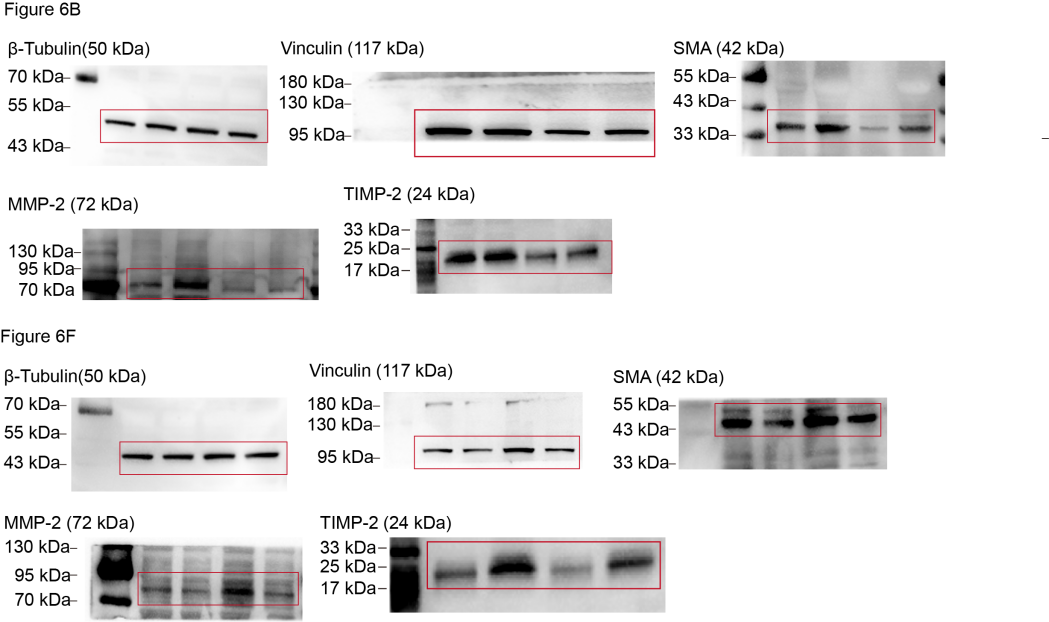
**
